# Multireceptor Analysis for Evaluating the Antidiabetic Efficacy of Karanjin: A Computational Approach

**DOI:** 10.1002/edm2.509

**Published:** 2024-07-09

**Authors:** Sagnik Nag, B. Stany, Shatakshi Mishra, Sunil Kumar, Sourav Mohanto, Mohammed Gulzar Ahmed, Bijo Mathew, Vetriselvan Subramaniyan

**Affiliations:** ^1^ Jeffrey Cheah School of Medicine and Health Sciences Monash University Malaysia Bandar Sunway Selangor Malaysia; ^2^ Department of Biomedical Sciences School of Bio‐Sciences & Technology (SBST), Vellore Institute of Technology (VIT) Vellore Tamil Nadu India; ^3^ Department of Pharmaceutical Chemistry Amrita School of Pharmacy, Amrita Vishwa Vidyapeetham, AIMS Health Science Campus Kochi India; ^4^ Department of Pharmaceutics, Yenepoya Pharmacy College & Research Centre Yenepoya (Deemed to Be University) Mangalore Karnataka India

**Keywords:** ADMET, diabetes, drug screening, in silico, insulin, Karanjin, MD simulation, molecular docking, phytocompounds

## Abstract

**Background:**

Diabetes mellitus, notably type 2, is a rising global health challenge, prompting the need for effective management strategies. Common medications such as metformin, insulin, repaglinide and sitagliptin can induce side effects like gastrointestinal disturbances, hypoglycemia, weight gain and specific organ risks. Plant‐derived therapies like Karanjin from *Pongamia pinnata* present promising alternatives due to their historical use, holistic health benefits and potentially fewer adverse effects. This study employs in silico analysis to explore Karanjin's interactions with diabetes‐associated receptors, aiming to unveil its therapeutic potential while addressing the limitations and side effects associated with conventional medications.

**Methodology:**

The research encompassed the selection of proteins from the Protein Data Bank (PDB), followed by structural refinement processes and optimization. Ligands such as Karanjin and standard drugs were retrieved from PubChem, followed by a comprehensive analysis of their ADMET profiling and pharmacokinetic properties. Protein–ligand interactions were evaluated through molecular docking using AutoDockTools 1.5.7, followed by the analysis of structural stability using coarse‐grained simulations with CABS Flex 2.0. Molecular dynamics simulations were performed using Desmond 7.2 and the OPLS4 force field to explore how Karanjin interacts with proteins over 100 nanoseconds, focusing on the dynamics and structural stability.

**Results:**

Karanjin, a phytochemical from *Pongamia pinnata*, shows superior drug candidate potential compared to common medications, offering advantages in efficacy and reduced side effects. It adheres to drug‐likeness criteria and exhibits optimal ADMET properties, including moderate solubility, high gastrointestinal absorption and blood–brain barrier penetration. Molecular docking revealed Karanjin's highest binding energy against receptor 3L2M (Pig pancreatic alpha‐amylase) at −9.1 kcal/mol, indicating strong efficacy potential. Molecular dynamics simulations confirmed stable ligand–protein complexes with minor fluctuations in RMSD and RMSF, suggesting robust interactions with receptors 3L2M.

**Conclusion:**

Karanjin demonstrates potential in pharmaceutical expansion for treating metabolic disorders such as diabetes, as supported by computational analysis. Prospects for Karanjin in pharmaceutical development include structural modifications for enhanced efficacy and safety. Nanoencapsulation may improve bioavailability and targeted delivery to pancreatic cells, while combination therapies could optimize treatment outcomes in diabetes management. Clinical trials and experimental studies are crucial to validate its potential as a novel therapeutic agent.

## Graphical abstract

The study evaluates Karanjin from *Pongamia pinnata* as a potential therapeutic for diabetes through in silico analysis. Karanjin showed promising ADMET properties, strong binding energies and stability in molecular dynamics simulation, especially with pancreatic alpha‐amylase (3L2M), suggesting its potential for diabetes management.

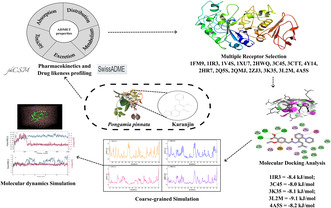

## 1 Introduction

Diabetes mellitus (DM) has emerged as a significant global health concern over recent decades, with its prevalence steadily increasing worldwide. The rising incidence of type 2 DM (T2DM) is primarily responsible for this epidemic. Understanding the epidemiological landscape of DM is crucial for effective public health interventions. Recent epidemiological data paint a concerning picture of the prevalence and incidence of DM. The burden of DM has surged significantly, with approximately 8.8% of the adult population diagnosed with the disease. Projections indicate that by 2040, nearly 10% of the global population aged 20–99 years will have DM, representing around 693 million individuals [1]. This escalation underscores the urgent need for preventive measures and effective management strategies. The incidence of DM varies by type, region, age and sex [2]. Type 1 DM (T1DM), often diagnosed in childhood or adolescence, is characterised by autoimmune destruction of pancreatic β‐cells [3, 4]. In contrast, T2DM, associated with insulin resistance and lifestyle factors, accounts for most cases [5, 6]. Gestational DM (GDM) poses risks to both mothers and infants during pregnancy [7]. The prevalence of DM increases with age, with most cases occurring in the fourth to seventh decades of life. In addition, men exhibit slightly higher prevalence rates compared to women, although this gender gap is expected to narrow in the future [8]. The urbanisation and income status are significant determinants of DM prevalence. Generally, urban centres report higher incidence rates compared to rural areas, reflecting lifestyle changes associated with urbanisation. Low‐income countries bear a disproportionately higher burden of DM, attributed to factors such as limited access to healthcare, rapid industrialisation and unhealthy lifestyle habits. Efforts to address these disparities must prioritise preventive strategies and equitable access to care. Primary prevention strategies play a pivotal role in mitigating the DM epidemic. Lifestyle modifications, including healthy dietary patterns, increased physical activity and avoidance of tobacco and alcohol, are cornerstone interventions. The Diabetes Prevention Program (DPP) has demonstrated the efficacy of intensive lifestyle interventions in reducing T2DM incidence [9, 10].

Diabetes diagnosis relies on traditional biomarkers such as glycated haemoglobin (HbA1c) [11], fasting plasma glucose (FPG) [12] and oral glucose tolerance test (OGTT) [12], supplemented by novel biomarkers including proteomic markers like glutamic acid decarboxylase (GAD) [13] and microRNAs (miRNAs), that is miR‐375 and miR‐21 [14, 15, 16, 17]. These biomarkers play a crucial role in early detection, risk stratification, monitoring disease progression and prognostication, enhancing personalised management strategies and improving patient outcomes.

A strategic approach to fully comprehending the intricate molecular landscape associated with diabetes is using several proteins as targets in molecular docking studies when examining possible medication candidates for treatment. Diabetes is characterised by the intricate interactions of several processes, such as insulin signalling, glucose metabolism and inflammatory responses [18, 19]. A comprehensive investigation of the various biological pathways connected to the illness is conceivable using numerous proteins as targets. A comprehensive investigation of possible medication interactions across a range of relevant targets is required due to the diverse isoforms of proteins, individual differences in protein expression and the multifactorial nature of diabetes [20]. The study strives to encounter possible medication candidates with broad efficacy or tailored specificity, which will aid in developing more effective and targeted medicines. The process uses 15 diabetes‐related proteins, selected based on a comprehensive literature review as a multireceptor approach, discussed in Table 1.

**TABLE 1:** Functional role of paramount receptors involved in DM.

SI. No.	Name of receptor	PDB ID	Role in diabetes	References
1.	Human RX alpha	1FM9	Heterodimers with PPARA, PPARA's activity in regulating fatty acid oxidation genes, diabetes pathogenesis, role in glucose/lipid metabolism and targets for antidiabetic therapies	[21]
2.	Phosphorylated insulin receptor tyrosine kinase	1IR3	Mediates glucose transport via the AKT/PKB pathway, regulating gluconeogenic/lipogenic enzymes, influencing diabetes pathogenesis, insulin resistance in diabetes disrupts these pathways, impairing glucose homeostasis	[22]
3.	Crystal structure of human glucokinase	1V4S	Glucose sensor in pancreatic beta cells, modulating insulin secretion in response to glucose levels, influences hepatic glucose usage and glycogen synthesis	[23]
4.	Crystal structure of the interface open conformation of tetrameric 11b HSD1	1XU7	Controls interconversion of glucocorticoids, influencing anti‐inflammatory responses, participates in aqueous humid secretion and bile acid metabolism, impacting metabolic homeostasis	[24]
5.	Peroxisome proliferator‐activated receptor agonists	2HWQ	Regulates adipocyte differentiation, glucose homeostasis, peroxisomal beta‐oxidation, controls P2 enhancers, suppressing inflammation and modulates circadian rhythms	[25]
6.	Human dipeptidyl peptidase IVCD26	3C45	T‐cell coactivation and lymphocyte‐epithelial adhesion	[26]
7.	Crystal complex of N‐terminal Human Maltase‐Glucoamylase	3CTT	Alpha‐(1,4) exo‐glucosidase, breaks down dietary starch into glucose in the small intestine, hydrolyses oligomaltoses, impacting postprandial blood glucose levels	[27]
8.	Structure of protein tyrosine phosphatase 1B	4Y14	Regulates the unfolded protein response and PERK activity, potentially influencing cell signalling pathways involved in glucose metabolism	[28]
9.	Insulin receptor (domains 1–3)	2HR7	Insulin's pleiotropic actions, activating PI3K‐AKT/PKB and Ras‐MAPK pathways, regulates glucose transport, lipogenic enzymes, protein synthesis and cell growth	[29]
10.	Crystal structure of PPARgamma	2Q5S	Regulates adipocyte differentiation, glucose homeostasis, fatty acid oxidation, suppression of inflammation and modulation of circadian rhythms	[30]
11.	Crystal structure of the N‐terminal subunit of human maltase	2QMJ	Breaks down starch into glucose in the small intestine, affecting postprandial blood glucose, hydrolyses oligomaltoses, influencing carbohydrate digestion and glycaemic control	[31]
12.	Isomerase domain of human glucose‐fructose‐6‐phosphate amidotransferase	2ZJ3	Controls glucose flux into the hexosamine pathway, crucial in diabetes for regulating protein glycosylation. Influences BMAL1 and CRY1 expression	[32]
13.	Crystal structure of human SIRT6	3K35	Regulates glucose metabolism, glycolysis and gluconeogenesis. Influences DNA repair, telomere maintenance and lipid metabolism	[33]
14.	Pig pancreatic alpha‐amylase	3L2M	Involved in carbohydrate digestion. Alteration in the activity of alpha‐amylase affects postprandial glucose levels	[34]
15.	Crystal structure of human DPP4	4A5S	T‐cell activation and coactivation, cell proliferation and NF‐Kappa‐B activation. Regulates cell adhesion, migration and proteolysis	[35]

The standard pharmacological therapeutics, that is Metformin, a first‐line T2DM medication, activates AMPK to reduce hepatic glucose production and enhance peripheral uptake. It lowers HbA1c by 1%–2% with minimal weight gain. Generally safe, it may cause mild gastrointestinal issues, with rare lactic acidosis in renal impairment. It also offers cardiovascular benefits [36]. Despite advancements like closed‐loop systems, barriers to adherence persist, necessitating patient education and support. Guidelines from organisations like the ADA and the EASD provide evidence‐based recommendations for insulin initiation, titration and monitoring, emphasising patient‐centred care to optimise outcomes and quality of life [37, 38]. An oral antidiabetic drug called Repaglinide increases the amount of insulin secreted by pancreatic beta cells, which helps regulate blood sugar. Its rapid onset and short duration of action make it suitable for mealtime dosing. Patient adherence and individualised dosing regimens are crucial for optimising its effectiveness in managing T2DM [37, 38]. Sitagliptin, an oral antidiabetic agent, works by inhibiting dipeptidyl peptidase‐4 (DPP‐4), thereby prolonging the action of incretin hormones. This leads to increased insulin secretion and decreased glucagon levels, helping to regulate blood glucose levels. Sitagliptin is typically taken once daily and is well‐tolerated, making it a valuable option for managing T2DM [39]. Several common side effects associated with these medications include gastrointestinal disturbances such as diarrhoea, nausea, vomiting with Metformin [40] and hypoglycaemia with insulin [41] and Repaglinide [42], manifesting as symptoms like sweating, trembling and dizziness. In addition, invasive site reactions may occur with insulin administration, while weight gain is a potential side effect of both insulin and Repaglinide [41, 42]. Additionally, upper respiratory tract infections and headaches are reported with Repaglinide [42] and Sitagliptin [43], respectively. Deficit in vitamin B12 may result from long‐term Metformin use [40], while Sitagliptin has been associated with rare cases of pancreatitis [43]. These side effects are based on clinical trials and postmarketing surveillance, emphasising the importance of individualised therapy and close monitoring.

Opting for plant‐based therapies offers several advantages, including fewer side effects, historical use in traditional medicine and holistic health benefits. These treatments often address root causes and promote overall wellness. Additionally, they support environmental sustainability and local communities involved in herbal cultivation and production [43]. *Pongamia pinnata* (L.) Pierre, a medium‐sized hairless tree from the Fabaceae family, is known as “Pongam” in Tamil, “Indian Beech” in English and “Karanja” in Hindi [44], widespread throughout India, primarily found in tidal forests. According to several investigations, the flowers of *P. pinnata* are reported to possess potential properties that combat high blood sugar levels and lipid peroxidation [45]. Studies have shown that administering aqueous (PPAE) and ethanolic (PPEE) extracts of *P. pinnata* leaves orally to alloxan‐induced diabetic rats significantly reduces plasma glucose levels [46]. This effect may stem from increasing insulin release from pancreatic β‐cells or freeing bound insulin, thus enhancing its efficacy. Additionally, the glucose‐lowering effect of PPAE and PPEE could be due to increased peripheral glucose utilisation [46]. Karanjin, a compound isolated from this plant, has been found to have hypoglycaemic effects in both normal and diabetic rats induced by alloxan. The ethanolic extract of *P. pinnata* fruits contains a chloroform‐soluble fraction containing pongamol and Karanjin, both of which have considerable glucose‐lowering properties [47]. These substances are thought to exert their antihyperglycaemic action by inhibiting protein tyrosine phosphatase 1B (PTPase‐1B), an essential component of insulin signalling and insulin resistance [47].

**FIGURE 1:**
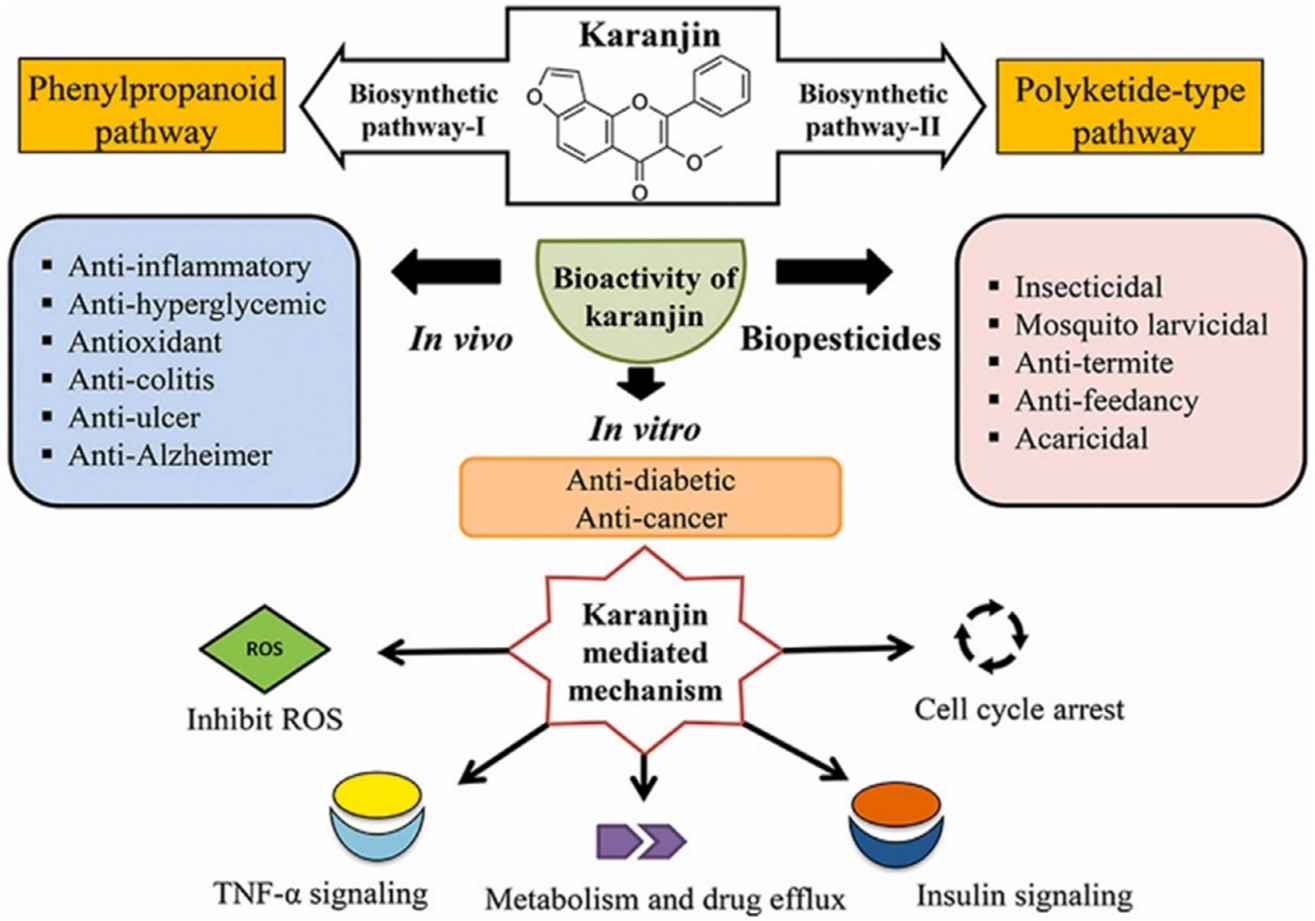
Schematic representation of the therapeutic activities of Karanjin, reproduced with permission from Ref., [49] Copyright 2021, Elsevier.

Karanjin, a compound abundant in *P. pinnata*, exhibits unique properties that differentiate it from other phytochemicals such as flavonoids and phenols. One significant advantage of Karanjin is its specific mechanism of action in combating hyperglycaemia, particularly through the inhibition of PTPase‐1B, a crucial regulator of insulin signalling and glucose metabolism [48, 49]. This mechanism has been demonstrated to be particularly effective in improving insulin sensitivity and reducing insulin resistance, making Karanjin a promising candidate for the management of DM [50]. Furthermore, Karanjin has been shown to possess potent hypoglycaemic activity in both normal and diabetic animal models, indicating its efficacy across a range of physiological conditions [47]. This broad spectrum of activity enhances its potential therapeutic value compared to other phytochemicals with more limited effects or mechanisms of action. Moreover, Karanjin's bioavailability and pharmacokinetic profile may also contribute to its superiority over other phytochemicals in the *P. pinnata* plant. Its ability to be fulfil the pharmacokinetics efficiently within the body could enhance its therapeutic effectiveness and reduce the risk of adverse effects [51]. Besides its role in managing diabetes, the phytocompound Karanjin is also implicated in diverse functions such as anti‐inflammatory [52], antioxidant [53], anti‐ulcer [53] and anti‐Alzheimer's [54] activities. Figure 1 briefly demonstrates the overall therapeutic activities exhibited by Karanjin.

This research presents innovative insights into the potential interactions between the phytoconstituent Karanjin derived from *Pongamia pinnata* and the identified receptors associated with diabetes, utilising in silico analysis. The study commenced with an in‐depth ADME (absorption, distribution, metabolism and excretion) analysis of both the standard drugs and Karanjin, followed by the retrieval of 15 receptors known to be involved in diabetes. Molecular docking analysis was then employed to assess the compatibility of Karanjin with these receptors, incorporating evaluations of docking scores and binding energies. The study utilised a multireceptor approach to encompass all the receptors involved in diabetes, aiming to discern the potential therapeutic nature and activity of Karanjin for diabetes management. The primary emphasis was placed on determining the binding energy, a crucial parameter for assessing the strength and stability of interactions. The analysis was benchmarked against the performance of existing drugs to ensure accuracy and reliability of the results. Moreover, coarse‐grained simulations were performed before the molecular dynamics (MD) simulations to study the dynamic behaviour of the protein with Karanjin at the atomic level. These simulations provided insights into intricate processes such as protein deviation, flexibility and conformational changes that are often challenging to observe through experimental methods alone. Coarse‐grained simulations were used to select the top two receptors for further investigation in MD simulations. With inadequate preclinical and clinical work on the therapeutic mechanisms of Karanjin, this study offers a pioneer comprehensive examination of its potential as a therapeutic agent for diabetes. The final selection of the best target receptor was based on MD simulations, providing a detailed understanding of the interactions between Karanjin and the chosen receptor. This multistep approach, from ADME analysis to MD simulations, enhances our understanding of Karanjin's molecular interactions and potential therapeutic applications for diabetes management.

## 2 Methods

### 2.1 Protein Preparation and Optimisation

The proteins were meticulously selected from the Protein Data Bank (PDB) (https://www.rcsb.org/), each identified with their respective names and PDB IDs, including RXRalpha (PDB ID: 1FM9), tyrosine kinase (PDB ID: 1IR3), glucokinase (PDB ID: 1V4S), tetrameric 11b HSD1 (PDB ID: 1XU7), peroxisome proliferator‐activated receptor (PDB ID: 2HWQ), dipeptidyl peptidase IV CD26 (PDB ID: 3C45), maltase‐glucoamylase (PDB ID: 3CTT), tyrosine phosphatase 1B (PDB ID: 4Y14), insulin receptor (PDB ID: 2HR7), PPARgamma (PDB ID: 2Q5S), maltase (PDB ID: 2QMJ), glucose‐fructose‐6‐phosphate amidotransferase (PDB ID: 2ZJ3), SIRT6 (PDB ID: 3K35), pig pancreatic alpha‐amylase (PDB ID: 3L2M) and DPP4 (PDB ID: 4A5S). Following this initial curation, a series of structural modifications were systematically applied to each protein. This process commenced with the removal of water molecules to ensure clarity and specificity in subsequent analyses. Next, hydrogen atoms were added to refine the molecular structure and accommodate optimal bonding configurations. Goldman charges were then incorporated to simulate the electrostatic interactions within the protein environment accurately. Additionally, any missing atoms were carefully replenished to restore integrity to the molecular framework. These procedures were methodically executed utilising advanced computational tools, notably AutoDockTools 1.5.7.

### 2.2 Retrieval of Ligands

The SDF files of the ligands, comprising Karanjin (PubChem ID: 100633), along with reference drugs like Metformin (PubChem ID: 4091), Repaglinide (PubChem ID: 65981) and Sitagliptin (PubChem ID: 4369359), were meticulously retrieved from PubChem (https://pubchem.ncbi.nlm.nih.gov/) [55]. These SDF files underwent a rigorous transformation process into pdbqt format utilising the sophisticated capabilities of the Open Babel server (https://www.cheminfo.org) [56]. Subsequently, employing precise computational techniques, the ligands were subjected to energy minimisation procedures, ensuring the refinement of their molecular structures to enhance accuracy and reliability for subsequent analyses.

### 2.3 ADMET Profiling and Pharmacokinetic Properties of the Ligands

The primary profiling of the ligands was performed based on Lipinski's rule of five and Veber's rules. The ADMET analysis was systematically performed for the provided ligands, encompassing the subheadings of Absorption, Distribution and Metabolism from the SwissADME webserver (http://www.swissadme.ch/index.php) [57, 58] followed by Excretion and Toxicity using pKCSM webserver (https://biosig.lab.uq.edu.au/pkcsm/) [59]. Each ligand's properties were evaluated within these categories. Specifically, factors such as bioavailability, gastrointestinal (GI) absorption, water solubility, blood–brain barrier (BBB) penetration, Cytochrome P450 2D6 (CYP2D6) inhibition/substrate status, renal OCT2 substrate status, oral rat acute toxicity LD50 (in mol/kg), total clearance (in log mL/min/kg), and AMES toxicity were meticulously examined [57, 59]. This comprehensive approach facilitated a thorough assessment of the ligands' pharmacokinetic and toxicological profiles, aiding in the comparison and identification of potential candidates for further investigation or therapeutic development.

### 2.4 Molecular Docking

The process is initiated with the determination of active sites linked with each protein by utilising the CASTp webserver (http://sts.bioe.uic.edu/castp/index.html?3igg), this facilitates the rigid docking. The study on molecular docking was conducted utilising AutoDockTools 1.5.7 with a methodically configured grid box setup. Analysis of the docking results and two‐dimensional interactions was performed using PyMOL [60] and BIOVIA Discovery Studio 2021 [61], respectively, encompassing both rigid docking and standard drug docking scenarios. This process involved a thorough examination of spatial arrangements and intermolecular interactions between the phytochemical ligand Karanjin and the established medications Metformin, Repaglinide and Sitagliptin. The primary aim was to thoroughly evaluate the structural aspects and binding modes of each docking strategy, enabling a detailed comparison and assessment of their results. This comprehensive evaluation helps in understanding the effectiveness of different docking approaches, providing deeper insights into the interactions and stability of protein‐ligand complexes.

### 2.5 Coarse‐Grained Simulation

The best binding energy complexes, which displayed satisfactory ADMET properties and docking outcomes, endured further screening by scrutinising their structural fluctuations using the CABS‐Flex 2.0 web server (https://biocomp.chem.uw.edu.pl/CABSflex2) [62]. During a 10‐ns period, CABS‐Flex 2.0 uses coarse‐grained CABS simulations to mimic the dynamic behaviour of a protein's secondary structure. Poisson‐Boltzmann/Generalised Born (PB/GB) molecular mechanics is used by the CABS‐Flex to assess the structural stability of the protein‐ligand combination. The simulation used the default restraint parameters and ran for 50 cycles using 50 trajectory frames. The resulting Root mean square fluctuation (RMSF) plot of the complexes attached to the ligand was compared to that of free proteins to examine variations in secondary structures. This analysis explores how ligand binding affects protein dynamics, offering insights into the mechanisms of action and stability of protein‐ligand complexes. By revealing conformational changes and stability shifts, it highlights the importance of dynamic studies for drug development, enhancing our understanding of protein function and interaction at the molecular level [62]. A drawback of coarse‐grained simulations, especially for short durations like 50 cycles, is their potential to sacrifice detailed atomic interactions for efficiency, necessitating further molecular dynamics simulations for a more comprehensive understanding of longer‐term dynamics and equilibrium behaviour.

### 2.6 Molecular Dynamic Simulation

The Desmond package, version 7.2, along with the OPLS4 force field, was employed in MD simulations to investigate Karanjin's lowest docking pose with two distinct proteins [22, 34, 63]. The simulations were executed on a Dell Inc. Precision 7820 Tower, operating Ubuntu 22.04.1 LTS 64‐bit. This system was equipped with an NVIDIA Corporation GP104GL (RTX A 4000) graphics processing unit and an Intel Xeon (R) Silver 4210R processor. Consistent with previous research, identical settings were used for the systems under investigation. The prior work contains more detailed information about the MD study, such as the solvent simulation box's size, shape, pressure control and temperature control parameters, as well as the treatment of long‐ and short‐range interactions [22, 34, 64]. To assess the correlations within the protein domains, Root Mean Square Deviation (RMSD) and RMSF analyses were conducted for each Cα atom for a 100 ns MD simulation [65, 66]. MD simulations are conducted to understand the physical movements and interactions of atoms and molecules over time. They provide insights into the structural dynamics, stability and functional mechanisms of biomolecules, aiding in drug design and the prediction of molecular behaviour under various conditions.

## 3 Results and Discussion

### 3.1 ADMET Profiling and Pharmacokinetic Properties of the Ligands

A comparison of the drug‐likeness properties among Karanjin, Metformin, Repaglinide and Sitagliptin reveals distinct characteristics. Karanjin and Metformin conform to Lipinski's Rule of Five and Veber's Rule, exhibiting optimal LogP, molecular weight (MW), hydrogen bond donors (NHD), hydrogen bond acceptors (NHA), total polar surface area (TPSA) and rotatable bonds (NRB) within acceptable limits as shown in Table 2. Sitagliptin also meets these criteria, indicating its potential as an orally bioavailable drug candidate. However, Repaglinide, while compliant with Lipinski's Rule of Five, exceeds Veber's Rule due to an elevated number of rotational bonds, suggesting a potential limitation in its bioavailability. Karanjin, unlike Repaglinide, does not violate Veber's rule, making it a better candidate based on this criterion. Veber's rule suggests good oral bioavailability when TPSA ≤140 Å^2^ and NRB ≤10. Repaglinide's violation can be overcome by modifying its structure to reduce the number of rotatable bonds. These findings underscore the favourable drug‐likeness of Karanjin, Metformin and Sitagliptin, positioning them as more promising candidates for further drug development.

**TABLE 2:** Drug‐likeness properties of the phytochemical and the standard drugs.

Phytochemicals	PubChem ID	Lipinski's rule					Veber's rule		
		NHD (≤10)	NHA (≤10)	LogP (≤5)	MW (g/mol) (≤500)	Violations	TPSA (Å^2^) (≤140)	NRB (≤10)	Violations
Karanjin	100,633	0	4	3.43	292.29	0	52.58	2	0
Metformin	4091	3	2	−0.89	129.16	0	91.49	2	0
Repaglinide	65,981	2	4	4.4	452.6	0	78.87	11	1
Sitagliptin	4,369,359	1	10	2.51	407.31	0	77.04	6	0

The ADMET properties of Karanjin, Metformin, Repaglinide and Sitagliptin were thoroughly examined. Karanjin and Sitagliptin exhibited moderately soluble characteristics, indicating a reasonable degree of water solubility. They both showed high GI absorption, suggesting efficient absorption upon oral administration. Karanjin was also identified as a substrate for CYP2D6 and renal OCT2, which could influence its metabolism and excretion, potentially leading to drug interactions and altered renal elimination as shown in Table 3. Metformin, on the other hand, displayed high water solubility, making it readily dissolvable in aqueous environments. Like Karanjin and Sitagliptin, it exhibited high GI absorption, indicating effective uptake from the gut. Metformin was not identified as a CYP2D6 or renal OCT2 substrate, suggesting a lower likelihood of drug interactions or altered renal excretion pathways. Repaglinide is presented with moderately soluble properties, falling between Karanjin, Sitagliptin and Metformin in terms of water solubility. It displayed high GI absorption, suggesting good bioavailability. Repaglinide has the highest total clearance among the four, indicating a faster elimination rate, which may affect its duration of action. Repaglinide does not exhibit AMES toxicity, suggesting a lower risk of mutagenicity. Sitagliptin demonstrated high water solubility, indicating good solubility in aqueous solutions. It exhibited high GI absorption, like the other compounds. Sitagliptin was not identified as a CYP2D6 or renal OCT2 substrate, indicating a lower risk of drug interactions or altered renal elimination pathways. Karanjin shows promise as a competitive candidate due to its moderately soluble characteristics, high GI absorption and identification as a substrate for CYP2D6 and renal OCT2, potentially offering advantages over standard drugs based on ADMET profiling.

**TABLE 3:** ADMET profile of Karanjin with the standard drugs.

Phytochemicals	Absorption		Distribution		Metabolism	Excretion			Toxicity
	Bioavailability	GI absorption	Water solubility	BBB penetration	CYP2D6 (Inhibitor/Substrate)	Renal OCT2 substrate	Oral rat acute toxicity LD50 (mol/kg)	Total clearance (log mL/min/kg)	AMES toxicity
Karanjin	0.55	High	Moderately soluble	Yes	Yes/No	Yes	2.448	0.353	Yes
Metformin	0.55	High	Highly soluble	No	No/No	No	2.453	0.1	Yes
Repaglinide	0.56	High	Moderately soluble	No	No/No	No	2.51	0.783	No
Sitagliptin	0.55	High	Soluble	No	No/No	No	2.732	0.474	No

Karanjin emerges as a superior drug candidate compared to Metformin, Repaglinide and Sitagliptin, being a phytochemical due to its unique natural origin, potentially offering advantages in efficacy, safety and reduced side effects. It adheres to Lipinski's and Veber's Rules with optimal LogP, molecular weight, hydrogen bond properties and rotatable bonds. In ADMET properties, Karanjin's moderate solubility, high GI absorption and ability to penetrate the BBB set it apart. While Metformin shares some properties, like solubility and GI absorption, it cannot cross the BBB. Repaglinide's fast clearance and inability to penetrate the BBB limit its potential. Sitagliptin, while meeting drug‐likeness criteria, also lacks BBB penetration. Karanjin's blend of favourable drug properties and unique ADMET profile positions it as a promising candidate for broad therapeutic effects in further drug development.

### 3.2 Molecular Docking Analysis

**FIGURE 2:**
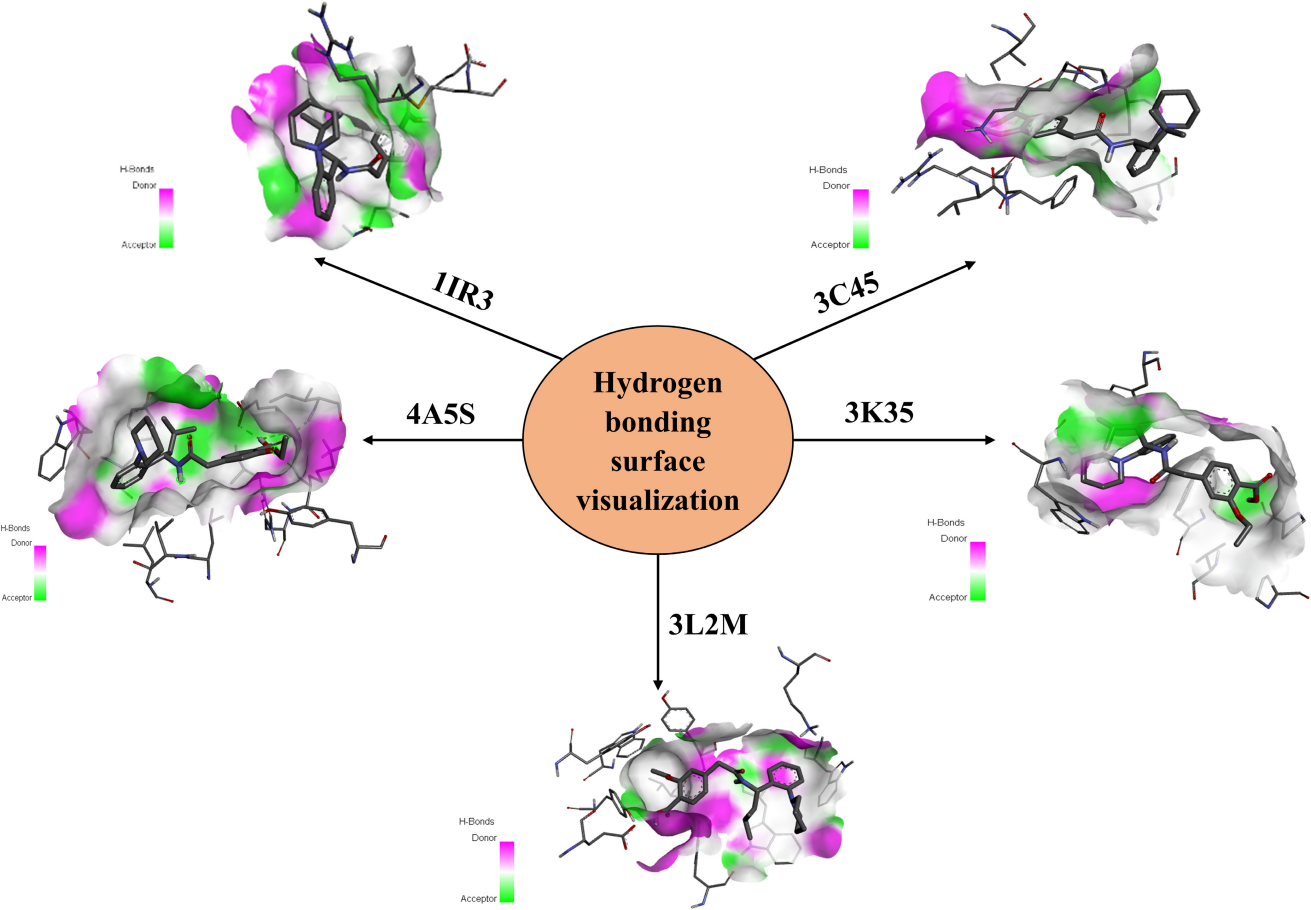
Visualisation of several receptors‐Karanjin binding via crucial hydrogen bond interactions.

The crystal structure of the 15 receptors obtained from PDB was used for docking analysis with Karanjin, Metformin, Repaglinide and Sitagliptin. Karanjin emerges with the highest binding energy of −9.1 kcal/mol against 3L2M, followed closely by 1IR3 at −8.4 kcal/mol, 4A5S at −8.2 kcal/mol, 3K35 at −8.1 kcal/mol and 3C45 at −8.0 kcal/mol. The binding energies of other receptors with Karanjin, and standard drugs are detailed in Table 4. The interaction observed between Karanjin and the top 5 binding energy receptors shows an average of 2 Hydrogen bonds in each as represented in Figure 2 wherein the purple colour present on the receptor showcases the hydrogen‐donating amino acid residues, while the green colour represents the hydrogen‐accepting residues.

**TABLE 4:** Molecular docking results of the 15 proteins with the phytochemicals.

SI. No	Protein	Grid box centre			Grid box dimension			Karanjin (Kcal/mol)	Metformin (Kcal/mol)	Repaglinide (Kcal/mol)	Sitagliptin (Kcal/mol)
		X	Y	Z	X	Y	Z				
1.	1FM9	7.471	−1.827	2.858	92	90	96	−7.2	−8.2	−6.9	−8.5
2.	1IR3	−24.01	28.485	11.284	100	62	98	−8.4	−8.6	−7.2	−8.1
3.	1V4S	19.644	3.092	73.223	80	80	78	−6.5	−7.2	−6.5	−7.4
4.	1XU7	−63.913	−81.138	−17.493	64	90	86	−7.2	−9.8	−8.3	−9.4
5.	2HR7	6.653	36.735	81.032	106	126	126	−7.8	−8.4	−7.7	−7.3
6.	2HWQ	−18.889	4.871	11.97	100	108	122	−7.8	−8.1	−8.4	−8.2
7.	2Q5S	15.135	16.632	11.92	80	78	96	−7.5	−9.4	−8.4	−9.1
8.	2QMJ	−25.298	9.471	−30.579	58	70	60	−7.5	−9.1	−8.0	−7.8
9.	2ZJ3	70.818	28.82	7.567	54	58	48	−7.0	−8.0	−7.3	−7.8
10.	3C45	−12.618	58.198	29.619	126	126	126	−8.0	−8.2	−7.1	−7.6
11.	3CTT	−3.536	3.445	34.534	54	62	60	−7.2	−8.8	−7.3	−8.0
12.	3K35	27.451	36.195	−46.898	82	78	60	−8.1	−8.6	−8.6	−8.8
13.	3L2M	34.922	36.819	55.078	72	64	54	−9.1	−8.7	−9.0	−8.8
14.	4A5S	11.772	22.321	56.686	118	126	126	−8.2	−8.8	−7.6	−8.7
15.	4Y14	11.948	−16.55	−18.407	82	66	72	−6.4	−6.8	−7.1	−7.2

**FIGURE 3:**
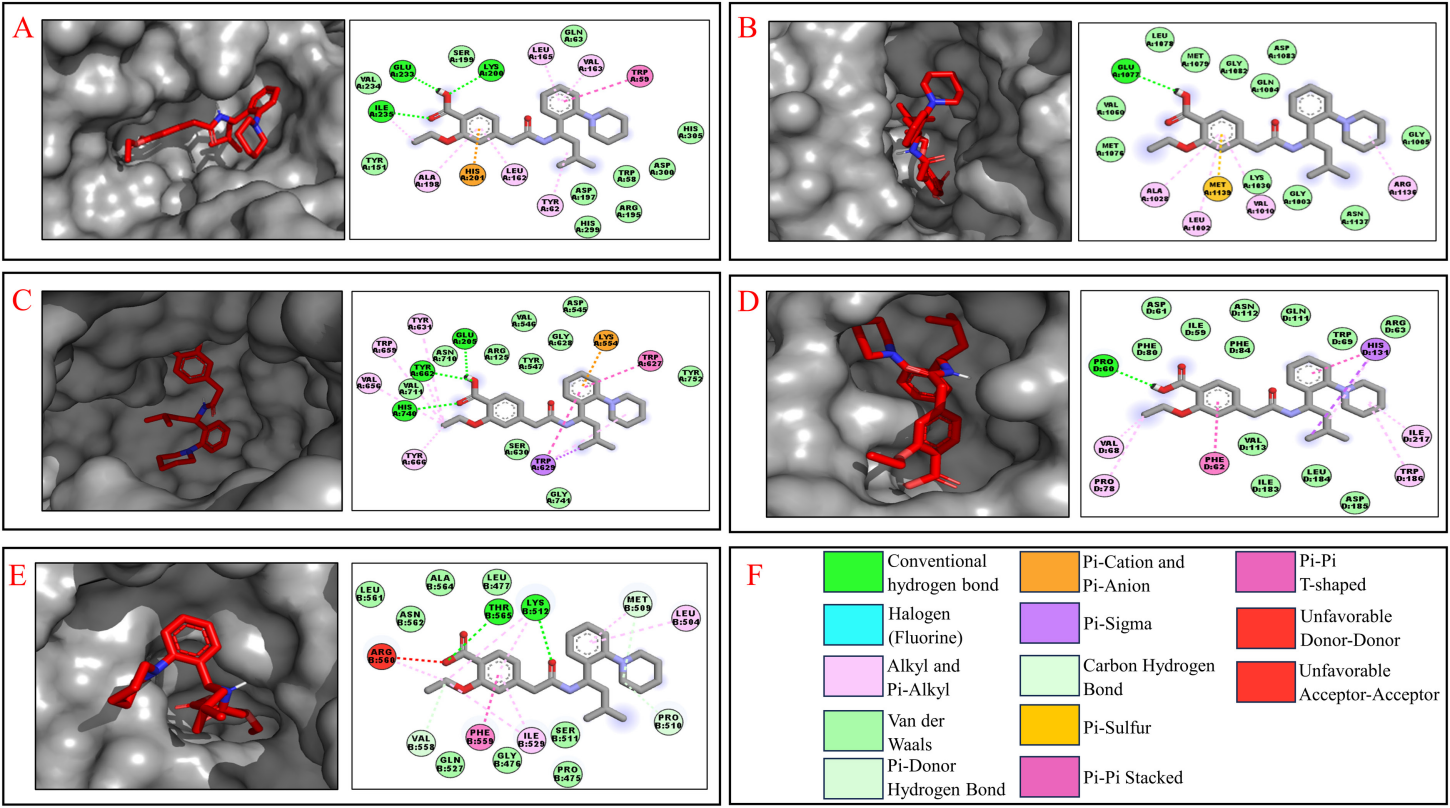
Molecular docking interaction of Karanjin with (A) 3L2M; (B) 1IR3; (C) 4A5S; (D) 3K35; (E) 3C45; (F) Legends of the interaction residues.

Karanjin forms hydrogen bonds with the LYS200, GLU233 and ILE235 residues of the 3L2M receptor (Figure 3A). It also interacts with the GLU1077 residue of the 1IR3 receptor (Figure 3B). In the 4A5S receptor, Karanjin forms bonds with the GLU205, TYR662 and HIS740 residues (Figure 3C). For the 3K35 receptor, it bonds with the PRO60 residue (Figure 3D). Finally, Karanjin establishes hydrogen bonding with the LYS512 and THR565 residues of the 3C45 receptor (Figure 3E). The interactions between Karanjin and other receptors, following the top 5 binding energies, are illustrated in Figure S1 and tabulated in Table S1. The interactions of Metformin, Repaglinide and Sitagliptin with the 15 receptors are illustrated in Figures S2–S4, respectively and are detailed in Table S1.

Karanjin demonstrates minimal differences in binding energies compared to standard drugs with the 1IR3, 4A5S, 3K35 and 3C45 receptors, while exhibiting higher binding energy with the 3L2M receptor than the standard drugs, indicating its potential competency in efficacy. This underscores the importance of further investigating these five proteins, selected from a pool of 15 based on their binding energies. This focused approach enriches our understanding of Karanjin's potential therapeutic properties and highlights the significance of exploring its interactions with these specific proteins.

### 3.3 Coarse Grained Simulations

**FIGURE 4:**
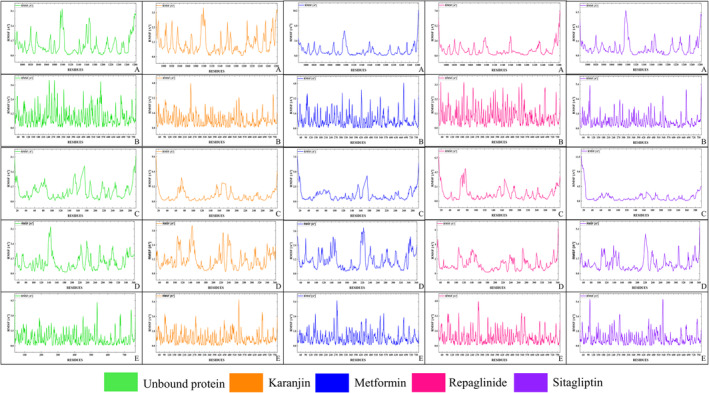
Pictorial representation of the RMSF plots of the Unbound and bound protein generated using CABS‐Flex. (A) 1IR3; (B) 3C45; (C) 3K35; (D) 3L2M; (E) 4A5S.

The top five proteins grounded on highest binding energies have undergone coarse‐grain simulations to determine their RMSF values (as shown in Table 5), which play a crucial role in assessing flexibility and conformational dynamics. This scrupulous approach enables a nuanced assessment of protein dynamics and flexibility across different binding scenarios, shedding light on how these proteins interact with various ligands such as Karanjin, Repaglinide, Sitagliptin and Metformin. Lower RMSF values generally signify increased stability within protein structures. This analysis is bifurcated into two main sections. Firstly, the focus is on determining the average RMSF values for each amino acid within the entire chain length, 3L2M‐Chain A, 1IR3‐Chain A, 3K35‐Chain D, 4A5S‐Chain A and 3C45‐Chain B, and then the analysis delves into the fluctuation of the amino acids that constitute the active sites. Upon analysing the entire chain length as depicted in Figure 4, 3C45 exhibits the lowest RMSF value with Karanjin (0.858 Å) compared to standard drugs, followed by Karanjin with 1IR3 (0.972 Å), Metformin with 3K35 (1.072 Å) and Metformin with 3L2M (1.125 Å). Notably, the fluctuation is higher when Karanjin and standard drugs are bound to 4A5S compared to the unbound protein. In active site residue analysis, Repaglinide exhibits the lowest fluctuation with 1IR3 (0.681 Å), closely followed by Karanjin (0.689 Å). Sitagliptin shows the least fluctuation with 3C45 (0.828 Å), followed by Repaglinide with 3K35 (1.201 Å) and Karanjin with 3L2M (0.792 Å). As observed with the entire chain length, fluctuation increases noticeably when Karanjin and standard drugs bind to 4A5S.

**TABLE 5:** RMSF values of the top 5 unbound and bound proteins along with the ligands.

Protein	RMSF (Å)					Fluctuation (Å)				
	Unbound	Karanjin	Metformin	Repaglinide	Sitagliptin	Unbound	Karanjin	Metformin	Repaglinide	Sitagliptin
1IR3	1.157	0.972	1.114	0.996	1.095	0.915	0.689	0.849	0.681	0.859
3C45	0.888	0.858	0.860	0.887	0.861	0.853	0.834	0.849	0.849	0.828
3K35	1.157	1.213	1.072	1.075	1.304	1.262	1.294	1.121	1.201	1.416
3L2M	1.276	1.141	1.125	1.276	1.146	1.229	0.792	0.819	1.08	0.871
4A5S	0.734	0.938	0.951	0.873	0.902	0.694	0.868	0.904	0.841	0.815

In this context, 3L2M emerges as a distinct case with significantly lower RMSF values around the active sites, especially evident when bound to Karanjin and a close difference with Repaglinide for the 1IR3 receptor and, therefore, is chosen for further analysis to check its entire stability. Based on the range of fluctuations observed, the other proteins are excluded from further study due to their higher fluctuation levels compared to 1IR3 and 3L2M. Since the current objective only examined protein fluctuation over 50 cycles and lacked detailed information, further comprehensive investigations into the specific molecular interactions at an atomic level of 3L2M and 1IR3 with Karanjin would be instrumental in elucidating their precise mechanisms of stabilisation by exposing the docked complexes to MD simulation.

### 3.4 Molecular Dynamics Simulation

**FIGURE 5:**
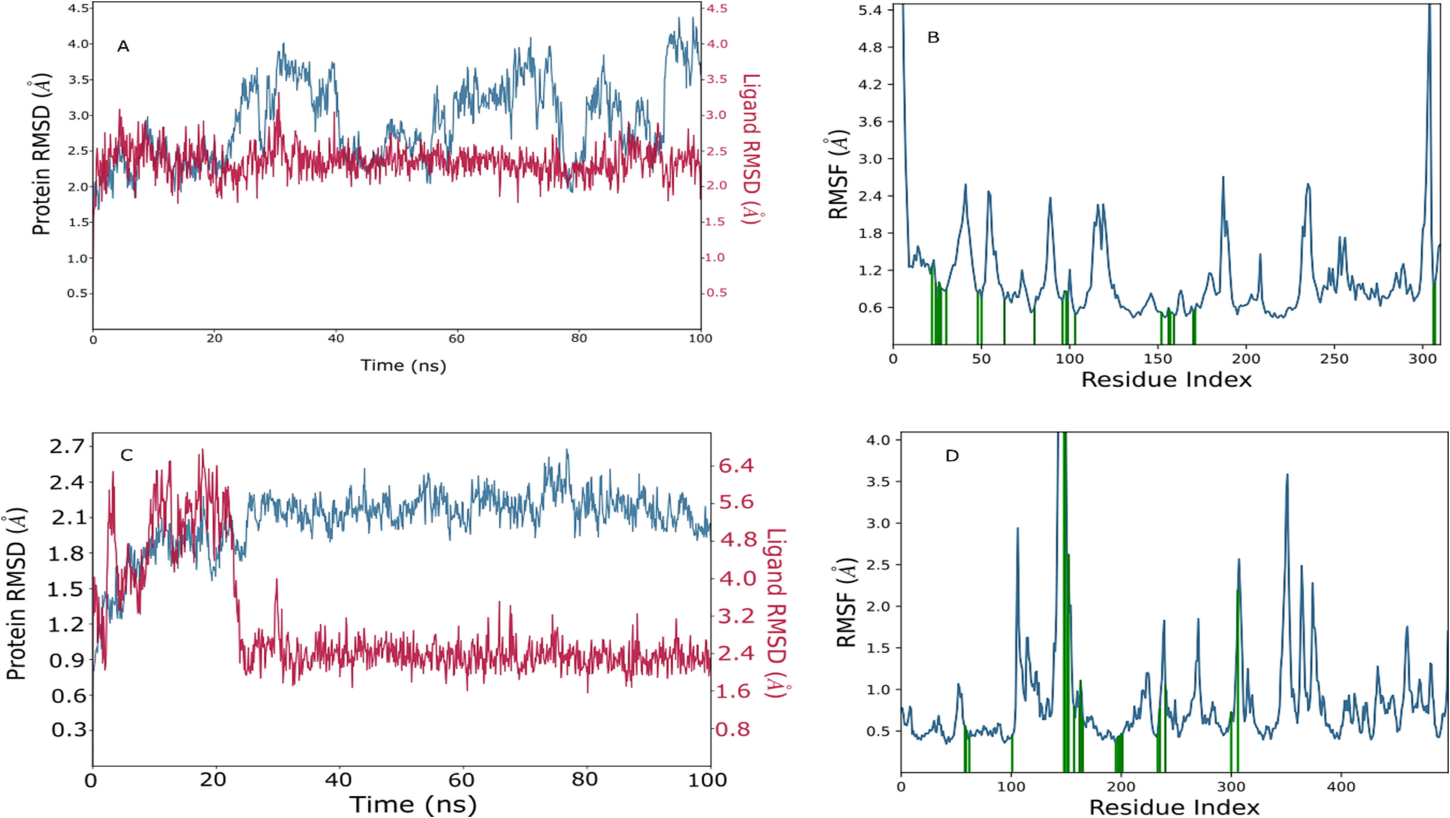
Analysis was conducted on the interaction of Karanjin with the complexes 1IR3 and 3L2M using molecular dynamics (MD) simulation. The RMSD plots for Karanjin with 1IR3 and 3L2M are represented by panels (A) and (C), respectively. Panels (B) and (D) depict the RMSF of individual amino acids for 1IR3 and 3L2M, respectively.

MD simulations were conducted on the Karanjin bound to 3L2M and 1IR3 proteins (depicted in Figure 5A–D) to assess complex stability and protein flexibility within biological environments. The resulting RMSD plots indicated stable ligand‐protein complexes, with RMSD values ranging from 0.9 to 2.7 Å for 3L2M Cα atoms and 1.6–4.5 Å for 1IR3. The ligand RMSD varied from 2.0 to 6.5 Å for 3L2M and 2.0–3.4 Å for 1IR3 concerning the proteins, showing minor fluctuations in the ligand RMSD until 10–20 ns, followed by stability (Figure 5C). 3L2M exhibited stable RMSD values, indicating a consistent protein‐ligand complex, while substantial fluctuations in both proteins and ligands for 1IR3 suggested instability. Analysis of protein flexibility via RMSF showed higher fluctuations in N‐terminal residues, with Karanjin interacting with amino acids 23 and 22 of 3L2M and 1IR3, respectively. These residues displayed RMSF values below 1 Å, indicating stable interactions (Figure 5B,D). The binding cavity of MAO‐B proteins showed very little conformational change, especially in the residues that interacted with Karanjin. The main chain and active site residues showed small variations in the RMSF plot, indicating strong binding with little conformational changes. The MD simulations revealed stable ligand‐protein complexes for both 3L2M and 1IR3, with 3L2M showing consistent stability throughout the simulation.

## 4 Conclusion

Karanjin emerges as a promising contender for pharmaceutical development, particularly in addressing metabolic disorders like diabetes. Its adherence to criteria for drug suitability, distinctive ADMET profile and robust interactions with target proteins such as 3L2M highlight its potential therapeutic effectiveness. Comparisons with established medications Metformin, Repaglinide and Sitagliptin emphasise Karanjin's unique advantages, particularly its potential for central nervous system effects and enhanced bioavailability. Moreover, as a natural compound, Karanjin offers a compelling option for drug development, potentially providing safer and more sustainable alternatives compared to synthetic compounds. In this stance, both the standard drug candidate and Karanjin, a phytochemical, have limitations that could be addressed through structural modifications using structural chemistry and cheminformatics. These chemical modifications can enhance Karanjin's therapeutic efficacy and mitigate its limitations, making it a more viable drug candidate. Alternative approaches include considering Karanjin as an adjuvant with standard drugs, exploring its potential in nanoformulations and using it as an analogue. Innovative drug delivery systems and formulation strategies for diabetes management could involve nanoencapsulation to improve bioavailability and target‐specific delivery to pancreatic cells. Additionally, developing sustained‐release formulations or combination therapies with synergistic agents could enhance its therapeutic efficacy, reduce dosing frequency and improve patient compliance and treatment outcomes. These strategies, along with further clinical trials and laboratory investigations, are imperative to validate and elaborate on the presented findings. These studies will provide a more comprehensive understanding of Karanjin's mechanisms of action, safety profile and potential adverse effects, paving the way for its utilisation as an innovative therapeutic agent in metabolic disorders and beyond. This work, serving as a pioneering effort in silico for diabetes management using Karanjin, holds significant potential. However, in silico studies have limitations, such as the inability to fully replicate the complexity of biological systems and the need for subsequent experimental validation to confirm findings. With limited in vivo and in vitro studies on this topic, this research stands to benefit the field of diabetes research by shedding light on various treatment strategies and offering insights into potential novel therapeutic avenues.

## Author Contributions

Sagnik Nag: conceptualisation, methodology, formal analysis and validation, writing–original draft preparation, writing–review and editing and overall supervision and project administration. B. Stany: methodology, formal analysis and validation, writing–original draft preparation and visualisation and Illustrations. Shatakshi Mishra: methodology, formal analysis and validation, writing–original draft preparation, visualisation and Illustrations. Sunil Kumar: methodology, formal analysis and validation. Sourav Mohanto: writing–review and editing. Mohammed Gulzar Ahmed: writing–review and editing, overall supervision and project administration. Bijo Mathew: overall supervision and project administration. Vetriselvan Subramaniyan: writing–review and editing, overall supervision and project administration. All authors have read and agreed to the published version of the manuscript.

## Conflicts of Interest

The authors declare no conflicts of interest.

## Supporting information


Appendix S1.

## Data Availability

The datasets produced throughout this study, and those analysed are readily obtained from the corresponding author upon the submission of a reasonable request.
