# Supplementary material for: Multireceptor Analysis for Evaluating the Antidiabetic Efficacy of Karanjin: A Computational Approach
Source: Endocrinol Diabetes Metab. 2024 Jul 9;7(4):e509. doi: 10.1002/edm2.509 (PMC11233261; doi:10.1002/edm2.509)
Supplement: Supplementary file 1 — Appendix S1. [file EDM2-7-e509-s001.docx]

**SUPPLEMENTARY MATERIALS**

**Table S1.** Molecular docking analysis of interacting residues of the 15 selected proteins with the 4 ligands.

| **Protein** | **Karanjin** | | **Metformin** | | **Repaglinide** | | **Sitagliptin** | |
| --- | --- | --- | --- | --- | --- | --- | --- | --- |
|  | **Hydrogen Interaction** | **Other Interactions** | **Hydrogen Interaction** | **Other Interactions** | **Hydrogen Interaction** | **Other Interactions** | **Hydrogen Interaction** | **Other Interactions** |
| **1FM9** | ASN377, GLN444 | ALA303, ASN306, GLU307, SER380, LYS381, ARG426, LEU430, LEU433, GLU434, PHE437, THR447, GLU448, GLN451, ALA457, PRO458 | ARG426, ASP441 | ALA303, ASN306, GLU307, PRO366, GLU369, PHE370, LYS373, ASN377, ASP379, SER380, LYS381, LEU430, LEU433, LYS438, GLN444, ILE445, PRO458 | - | VAL372, LYS373, ALA376, LEU377, PRO378, ASP379, PRO386, ALA387, GLU390, ARG393, TYR397, LYS434, GLN437, LYS438 | - | LEU255, GLU259, ILE262, PRO269, LEU270, ILE281, GLY284, CYS285, PHE287, ARG288, ILE341, SER342, MET348 |
| **1IR3** | GLU1077 | LEU1002, GLY1003, GLN1004, GLY1005, VAL1010, ALA1028, LYS1030, VAL1060, MET1076, LEU1078, MET1079, GLY1082, ASP1083, ARG1136, ASN1137, MET1139 | LYS1030, ARG1136 | LEU1002, GLY1003, SER1006, VAL1010, ALA1028, MET1076, GLU1077, LEU1078, MET1079, GLY1082, ASN1137, MET1139, GLY1149, ASP1150 | MET1079 | LEU1002, GLY1003, GLN1004, GLY1005, SER1006, VAL1010, ALA1028, LYS1030, GLU1047, ASP1050, MET1076, LEU1078, GLY1082, ASP1083, ARG1136, ASN1137, MET1139 | MET1079 | LEU1002, GLY1003, GLY1005, VAL1010, ALA1028, LYS1030, VAL1060, MET1076, GLU1077, LEU1078, GLY1082, ASP1083, ASN1137, MET1139, ASP1150 |
| **1V4S** | ASP78, GLY81, LYS169, ASN409 | GLY80, ASN83, ARG85, MET107, THR149, SER151, ASP205, THR209, ILE225, GLY227, THR228, GLY410, SER411, LYS414 LEU415, GLU443, GLY444, SER445, | ARG85, THR228, ASP409, SER445 | ASP78, GLY81, SER151, LYS169, ASP205, ILE225, GLY227, GLY229, GLY410, SER411, LYS414, LEU415 | GLY81, ASP409 | ASP78, GLY80, ASN83, ARG85, MET107, SER151, LYS169, ASP205, ILE225, GLY227, THR228, GLY410, SER411, LYS414, GLU443, GLY444, SER445 | ARG369, ARG422 | PHE23, GLN24, LEU25, GLU372, SER373, THR376, HIS380 |
| **1XU7** | - | LEU126, LEU171, TYR177, LEU217, VAL231, VAL227, SER228, GLN234, ASP259, THR264, LEU276, LEU279, TYR280 | ILE46, TYR183, LEU215 | LYS44, GLY45, GLY47, ASN119, ILE121, LEU126, SER169, SER170, LEU171, ALA172, TYR177, VAL180, GLY216, LEU217, THR220, THR222, ALA223, TYR280 | TYR177 | ILE46, THR124, LEU126, SER170, LEU171, ALA172, VAL180, TYR183, LEU215, GLY216, LEU217, ILE218, ALA223, VAL227, VAL231, GLN234, ASP259, SER260, LEU276, TYR280 | ASN119, THR220, ALA223 | ILE46, ILE121, THR124, VAL168, SER170, ALA172, VAL180, TYR183, LYS187, THR222, ALA226 |
| **2HR7** | GLY5 | PRO4, GLU6, VAL7, SER27, VAL28, MET56, CYS225, VAL226, ALA227, CYS228, TYR232, LEU233, ASP234, GLY235, TRP251, ARG252, CYS253 | GLU70, ASP74, LYS102 | PHE46, ARG47, LEU69, SER71, LYS73, LEU75, GLU103, CYS126, TYR127, LYS149 | PRO4, GLY5, CYS228, TYR232 | GLU6, VAL7, SER27, VAL28, CYS225, VAL226, ALA227, ASP234, GLY235, CYS237, TRP251, ARG252 | - | PRO4, SER27, VAL28, ILE55, SER217, THR223, CYS225, VAL226, ALA227, TYR232, GLY235, TRP251 |
| **2HWQ** | CYS285, SER342 | ILE249, LEU255, GLY258, GLU259, ILE262, GLN271, ARG280, ILE281, GLY284, PHE287, ARG288, LEU330, VAL339, ILE341, MET348, LEU353, MET364 | GLY284, CYS285 | MET248, LEU255, GLU259, ILE281, ARG280, PHE287, ARG288, SER289, ALA292, ILE326, LEU330, LEU333, ILE341, SER342 | - | LEU255, ARG280, ILE281, GLY284, CYS285, PHE287, ARG288, SER289, ILE326, TYR327, LEU330, MET334, VAL339, ILE341, SER342, MET348, HIS449, LEU353, MET364, LYS367 | CYS285 | ILE281, ARG288, LEU330, LEU333, MET334, VAL339, ILE341, SER342, GLU343, MET348, LEU353, MET364, LYS367 |
| **2Q5S** | LYS232, ASP243, LYS244, SER245 | ILE236, PRO246, PHE247, GLY344, GLN345 | LEU228, ARG288, LEU340 | PHE226, PRO227, ILE281, GLY284, CYS285, ALA292, GLU295, ILE326, MET329, LEU330, LEU333, VAL339, ILE341, GLU343, SER342, MET348 | LYS232, LYS244, SER245 | ILE236, ASP243, PRO246, PHE247, GLY344, GLN345 | - | ILE281, GLY284, CYS285, PHE287, ARG288, ILE326, LEU330, LEU333, VAL339, ILE341, MET348, MET364 |
| **2QMJ** | THR269, GLN272 | GLU271, GLN275, TYR636, LEU640, ARG643, ARG647, ASP649, ARG653, HIS657, GLU658, TYR660, PRO676, TYR733, LYS765, GLY766, GLU767, GLU788 | ARG647 | THR269, GLU271, TYR636, THR639, LEU640, ARG643, ARG653, HIS657, GLU658, TYR660, PRO676, ARG730, TYR733, ILE734, PRO736, LYS765, GLY766, GLU767 | ARG653, GLU767 | GLU271, GLN275, THR269, TYR636, LEU640, ARG643, ARG647, ASP649, GLU658, TYR660, PRO676, TYR733, ILE734, PRO736, LYS765, GLY766 | LYS776, VAL779 | ALA285, LEU286, LYS513, ARG520, LYS534, PHE535, HIS645, ASP777, THR778, ALA780 |
| **2ZJ3** | SER420, GLN421, SER422 | CYS373, GLY374, THR375, SER376, GLY423, THR425, VAL471, SER473, LEU556, GLU560, LEU673, ALA674, LYS675, SER676, VAL677 | SER376, GLN421, SER422, ALA674, LYS675 | CYS373, GLY374, THR375, SER420, GLY423, THR425, VAL471, SER473, LEU556, GLU560, LEU673, SER676, VAL677 | SER376, GLN421, SER422 | CYS373, GLY374, THR375, LEU399, SER401, LEU419, SER420, GLY423, GLU424, THR425, ASP427, SER473, GLN480, LEU673, LYS675, SER676, VAL677 | GLN421, THR425, VAL677 | THR375, SER376, SER420, SER422, SER473, GLN480, GLY534, LEU556, GLU560, SER676 |
| **3C45** | LYS512, THR565 | PRO475, GLY476, LEU477, LEU504, MET509, PRO510, SER511, GLN527, ILE529, VAL558, PHE559, ARG560, LEU561, ASN562, ALA564 | SER106, ILE107, VAL155, LYS463 | ARG61, TRP62, ILE63, LEU69, SER108, PRO109, LEU115, GLU117, TYR128, TYR132, THR156, TRP157 | SER376, THR350, THR351, ASP588 | ILE346, GLU347, MET348, SER349, GLY352, ILE375, ASN377, GLU378, GLY380, PHE387, CYS394, PHE396, HIS592 | GLN731 | LEU701, ASP729, GLN731, HIS754, HIS757, PHE758, GLN761 |
| **3CTT** | ARG647, HIS657, GLU658 | ASG269, GLU271, TYR636, ARG643, ASP649, ARG653, TYR660, PRO676, TYR733, ILE734, LYS765, GLY766, GLU767, GLU788 | ARG547 | THR269, GLU271, TYR636, THR639, LEU640, ARG643, ARG653, HIS657, GLU658, TYR660, PRO676, ARG730, TYR733, ILE734, PHE735, PRO736, LYS765, GLY766, GLU767 | GLN275 | THR269, GLU271, GLN272, TYR636, LEU640, ARG643, ARG647, ASP649, ARG653, HIS657, GLU658, TYR660, PRO676, TYR733, LYS765, GLY766, GLU767 | ARG647 | TYR636, THR639, LEU640, ARG643, ASP649, ARG653, PRO676, TYR733, ILE734, PRO736, GLU766, GLU767 |
| **3K35** | PRO60 | ILE59, ASP61, PHE62, ARG63, VAL68, TRP69, PRO78, PHE80, PHE84, GLN111, ASN112, VAL113, HIS131, ILE183, LEU184, ASP185, TRP186, ILE217 | GLY52, HIS66, LYS79, LEU239 | ASP61, GLU20, THR55, PRO60, GLY64, PRO65, GLY67, MET71, PRO78, ASP81, GLY212, GLY240, ASN238, TYR255, VAL256 | GLU138 | ALA140, LYS141, LYS143, GLN145, LEU157, ARG180, ASP181, THR182, LEU184, SER189, PRO191, ASP192, ARG193, ASP199, GLU200, ARG203 | - | GLY50, ALA51, PHE62, ARG63, TRP69, GLN111, ASN112, VAL113, HIS131, TRP186, THR213, SER214, LEU215, GLN216, ILE217 |
| **3L2M** | LYS200, GLU233, ILE235 | TRP58, TRP59, TYR62, GLN63, TYR151, LEU162, VAL163, LEU165, ARG195, ASP197, ALA198, SER199, HIS201, VAL234, HIS299, ASN300, HIS305 | LYS200, ILE235, ASP300 | TRP58, TRP59, TYR62, GLN63, HIS101, TYR151, LEU162, VAL163, LEU165, ASP197, ALA198, HIS201, GLU233 | GLU233, ILE235 | TRP58, TRP59, TYR62, GLN63, TYR151, LEU162, VAL163, LEU165, ARG195, ASN197, ALA198, SER199, LYS200, HIS201, VAL234, HIS299, ASP300, HIS305 | - | TRP58, TRP59, TYR62, GLN63, HIS101, LEU162, VAL163, LEU165, ARG195, ASP197, GLU233, HIS299, ASP300, HIS305 |
| **4A5S** | GLU205, TYR662, HIS740 | ARG125, LYS554, ASP545, VAL546, TYR547, TRP627, GLY628, TRP629, SER630, TYR631, VAL656, TRP659, TYR666, ASN710, VAL711, GLY741, TYR752 | GLU205, GLU206, TYR547, ARG669, HIS740 | ARG125, PHE357, ARG358, GLY549, TRP629, SER630, TYR631, VAL656, TRP659, TYR662, TYR666, TYR670, ASN710, VAL711 | LYS554 | GLN527, ILE529, ASP545, TYR547, GLN553, ASP556, ARG560, VAL546, ASN562, ALA564, SER577, TRP627, GLY628, TRP629, SER630, TYR631, GLY632, TYR752, GLN741 | - | ARG125, GLU205, TYR547, TRP629, SER630, TYR631, GLY632, VAL656, TRP659, TYR662, TYR666, VAL711, HIS740 |
| **4Y14** | HIS54, LYS58, HIS60, GLN102 | SER55, ARG56, ILE57, LEU71, LYS73, LYS128, GLU129, GLU130, PHE256 | ASP48, ASP181 | TYR46, VAL49, LYS116, LYS120, PHE182, SER216, ALA217, ILE219, GLY220, ARG221, GLN262 | PHE182, ARG221 | TYR46, ASP48, VAL49, LYS120, ASP181, CYS215, SER216, ALA217, GLY218, ILE219, GLY220, GLN262, GLN266, | - | HIS25, GLU26, SER28, ASP29, PHE30, LYS141, THR143, ILE145, GLU159, GLU161, GLN166, THR168, LYS255 |


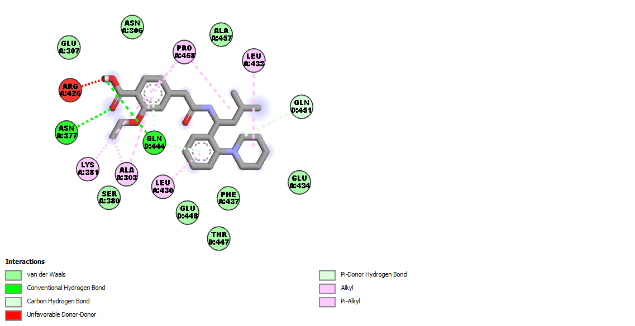

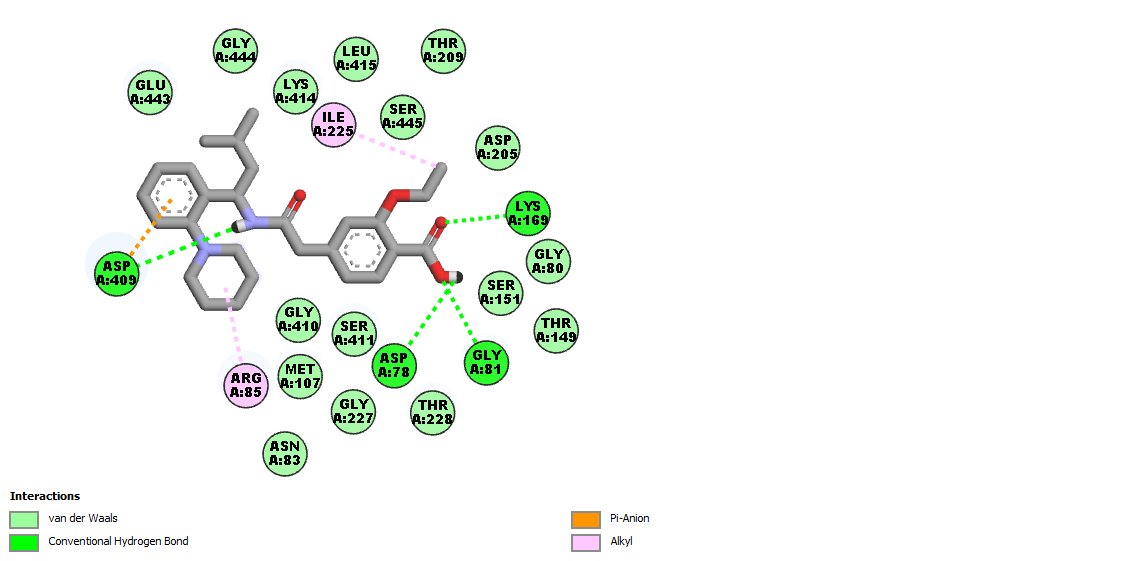

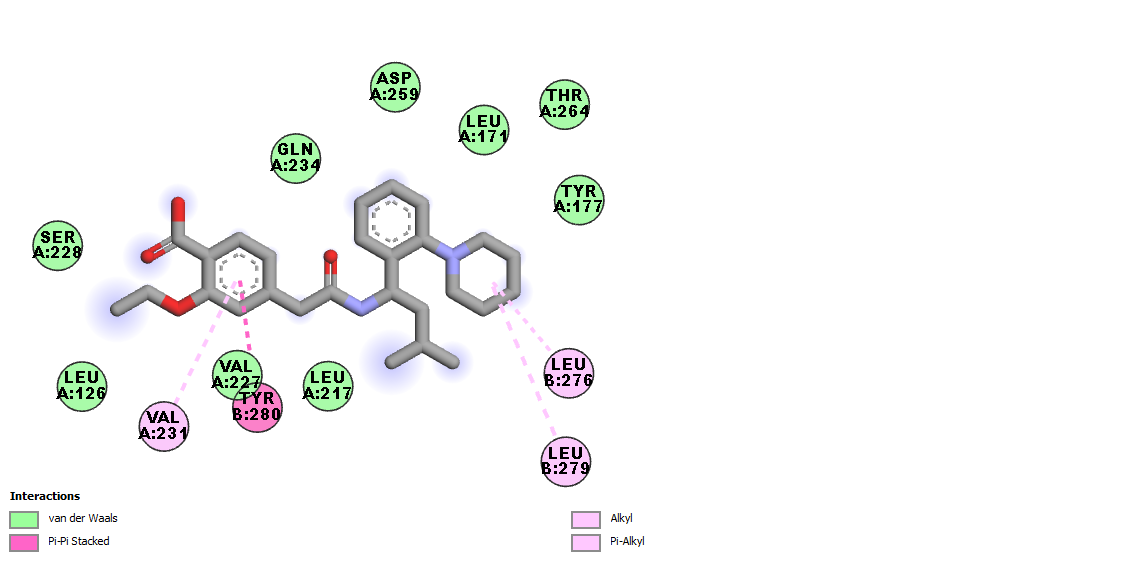

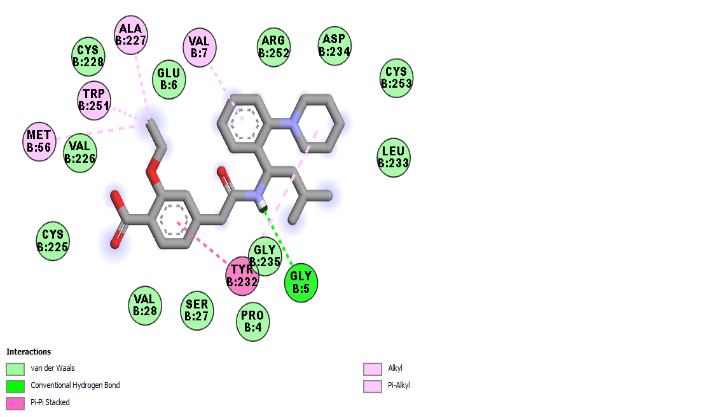

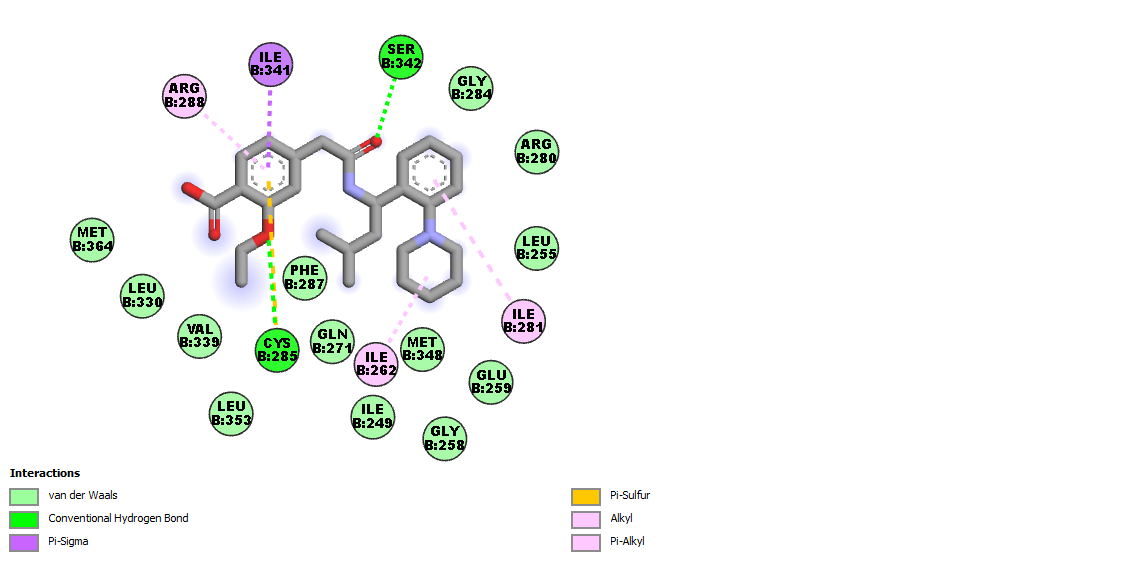

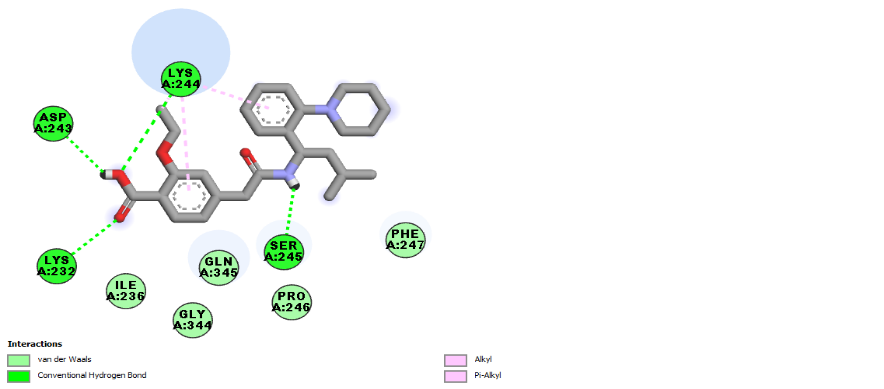

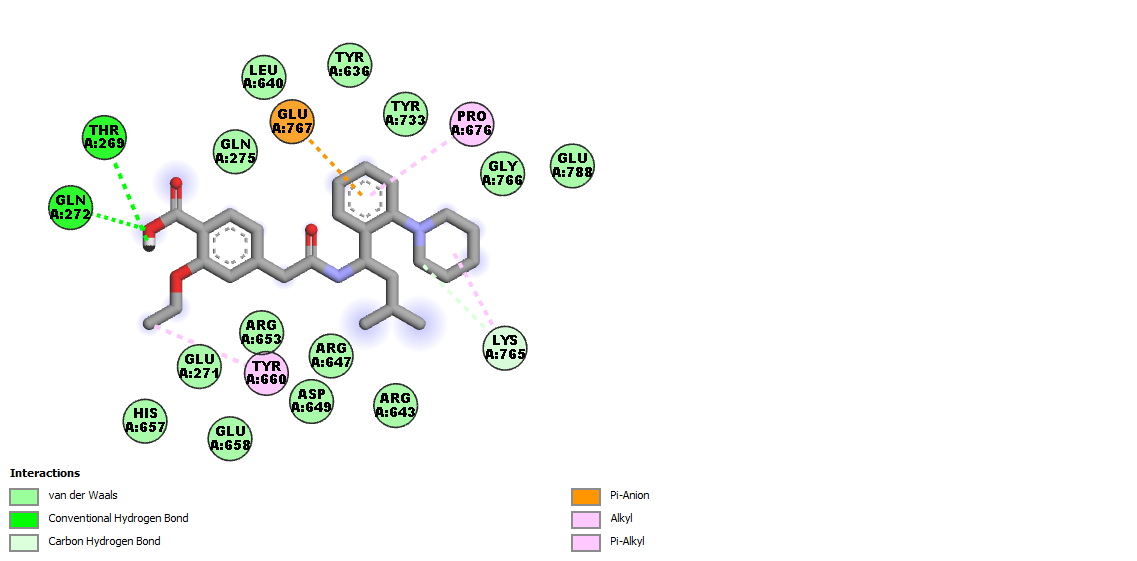

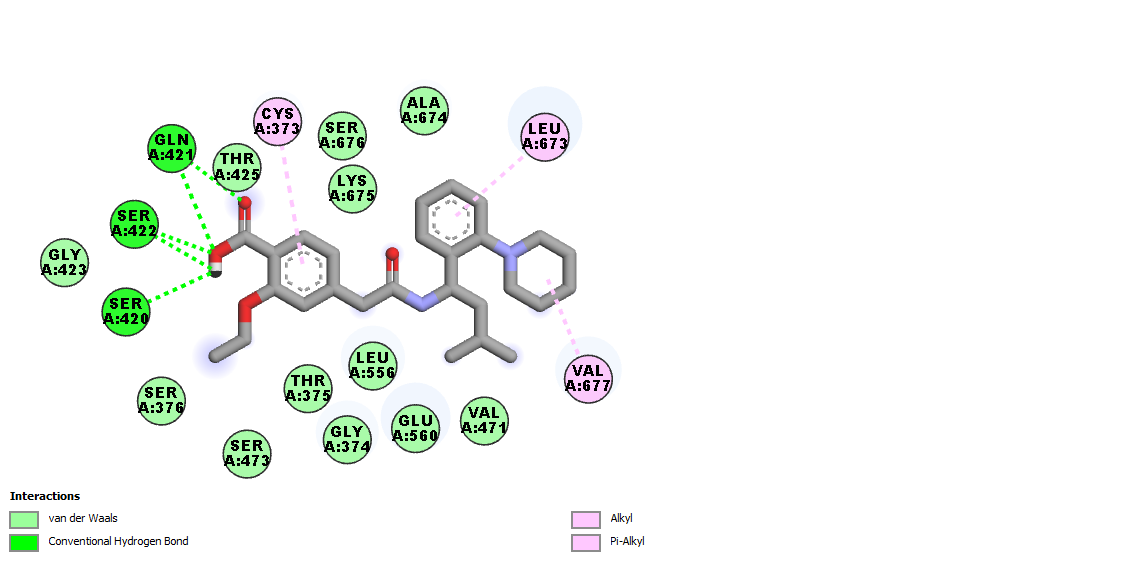

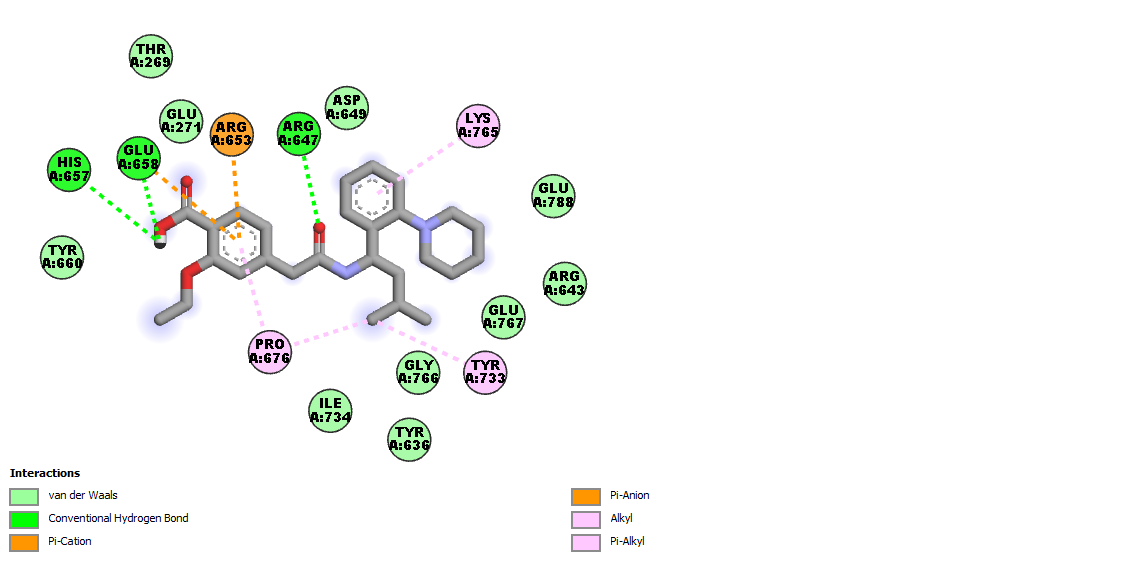

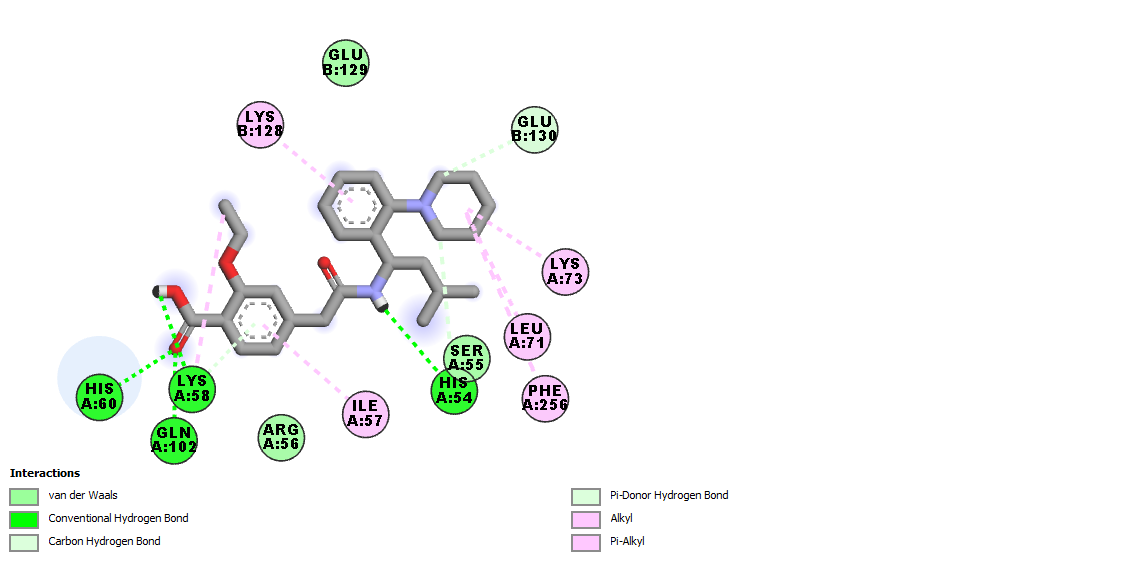


A

B

C

D

E

F

G

H

I

J

**
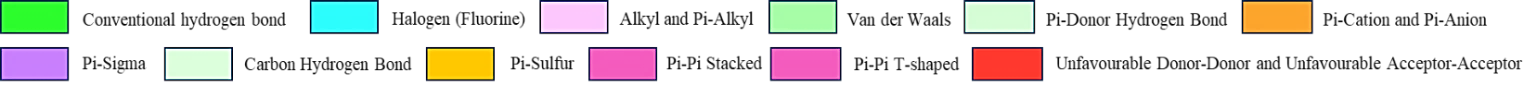
**

**Figure S1** Molecular docking interaction of Karanjin with (**A**) 1FM9; (**B**) 1V4S; (**C**) 1XU7; (**D**) 2HR7; (**E**) 2HWQ; (**F**) 2Q5S; (**G**) 2QMJ; (**H**) 2ZJ3; (**I**) 3CTT; (**J**) 4Y14.


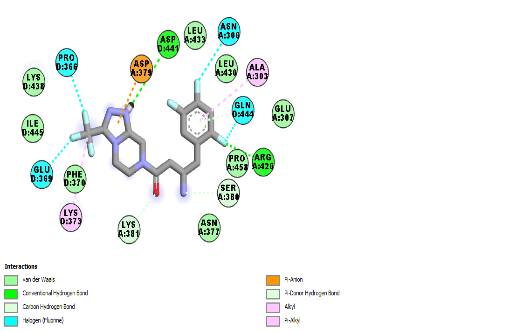

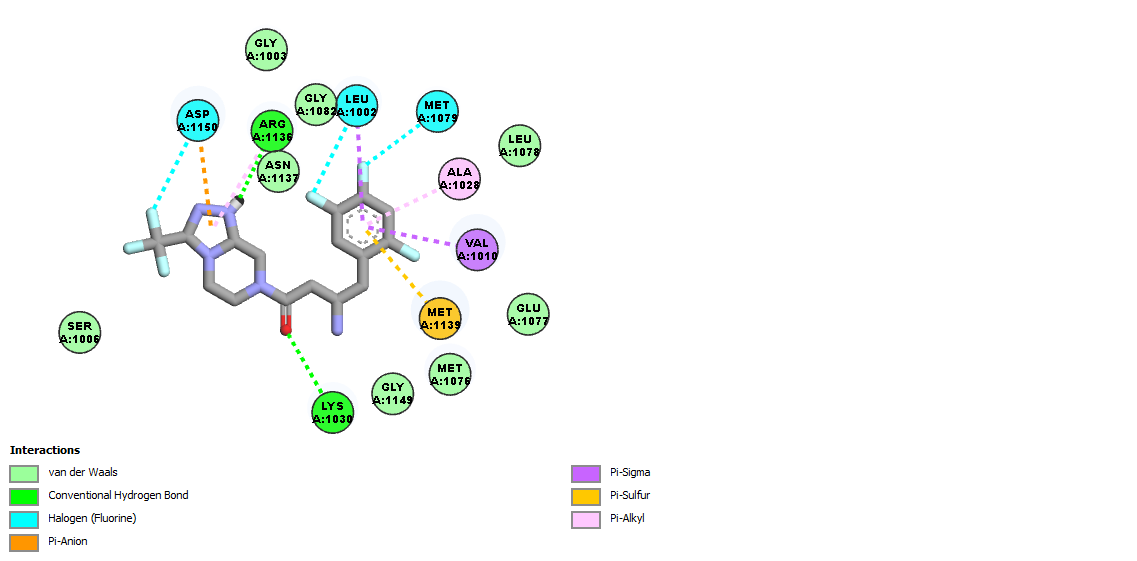

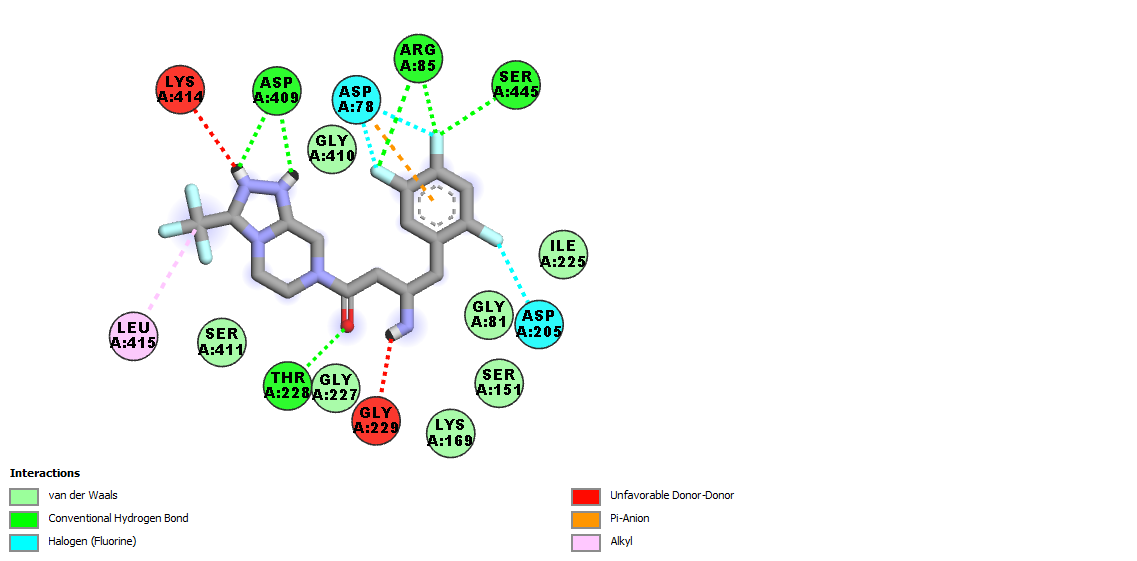

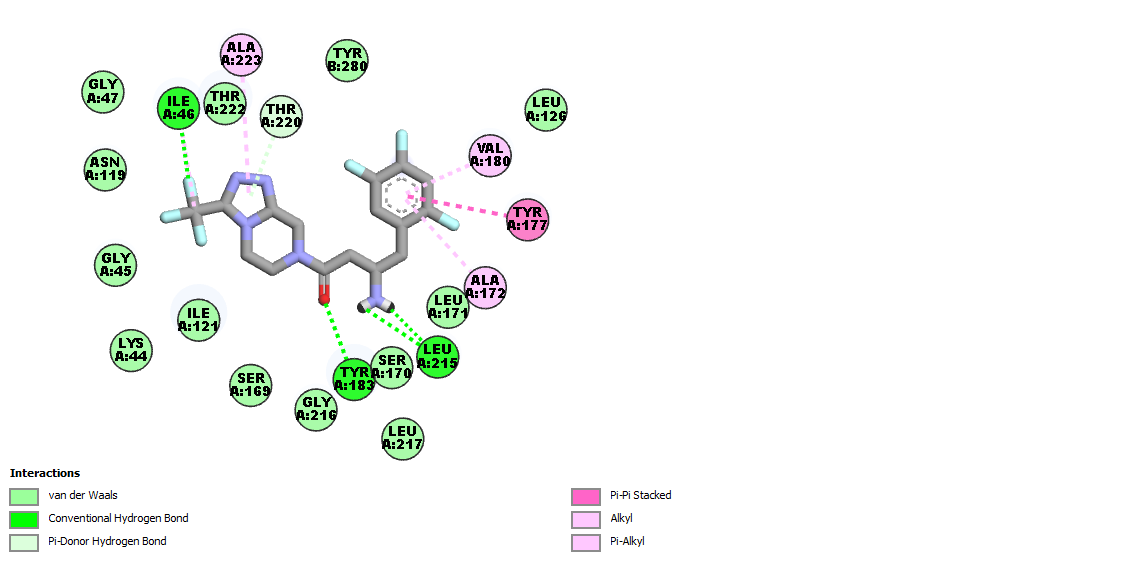

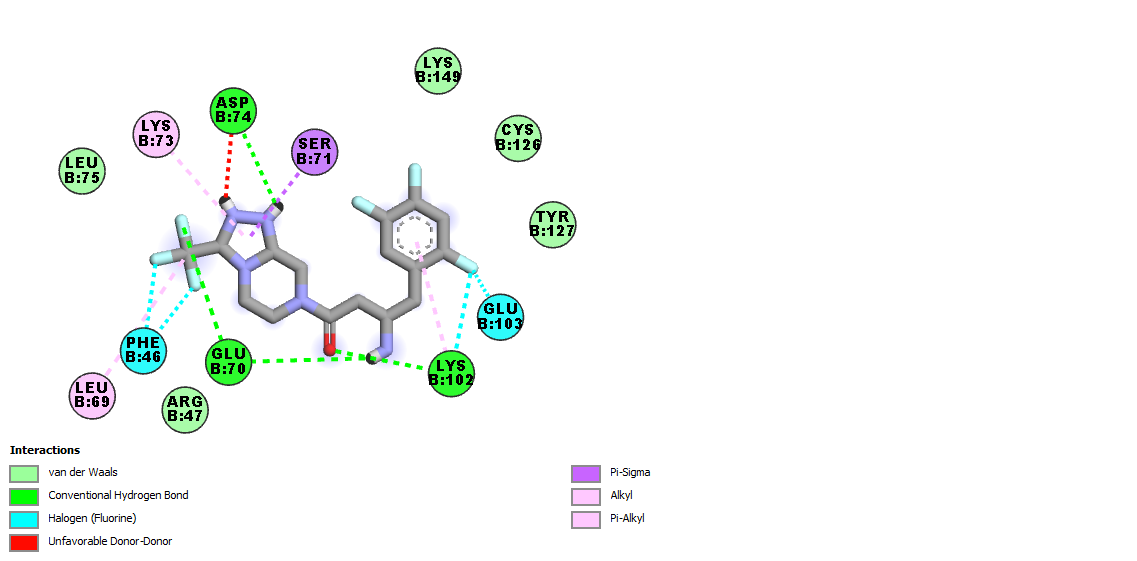

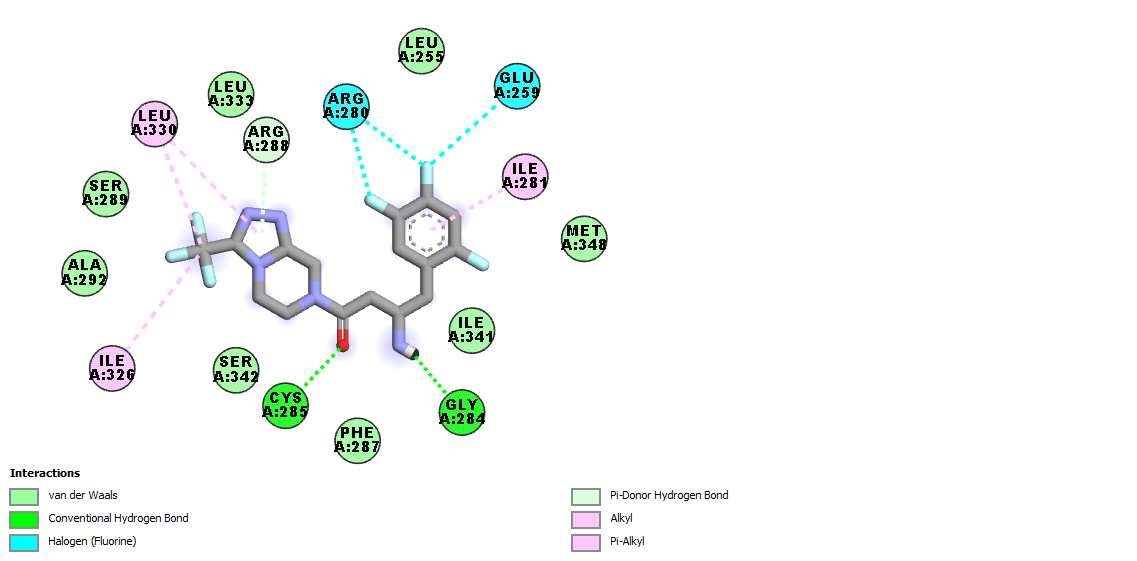

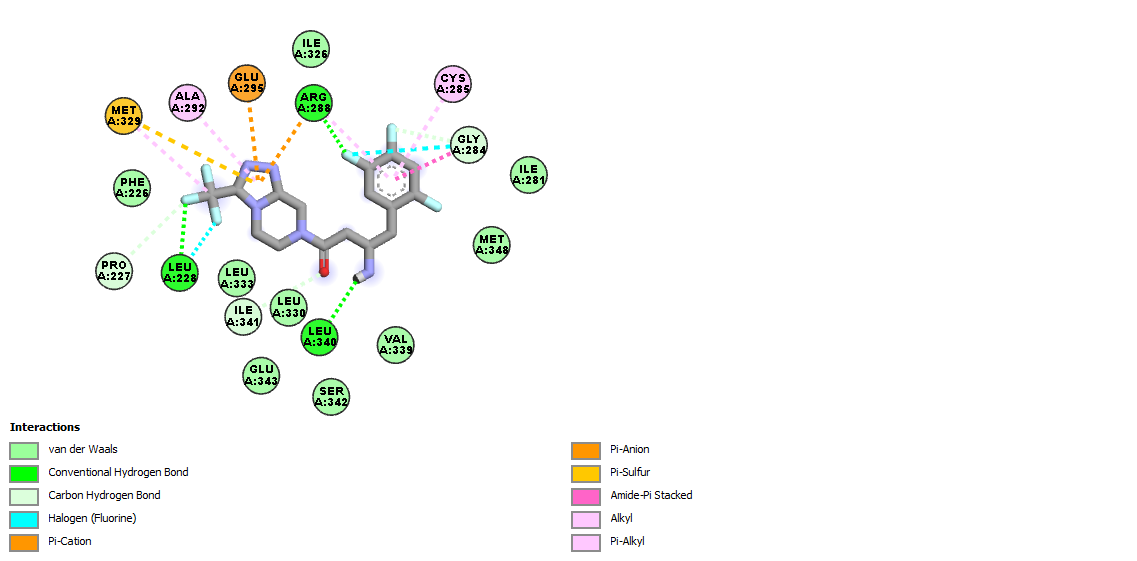

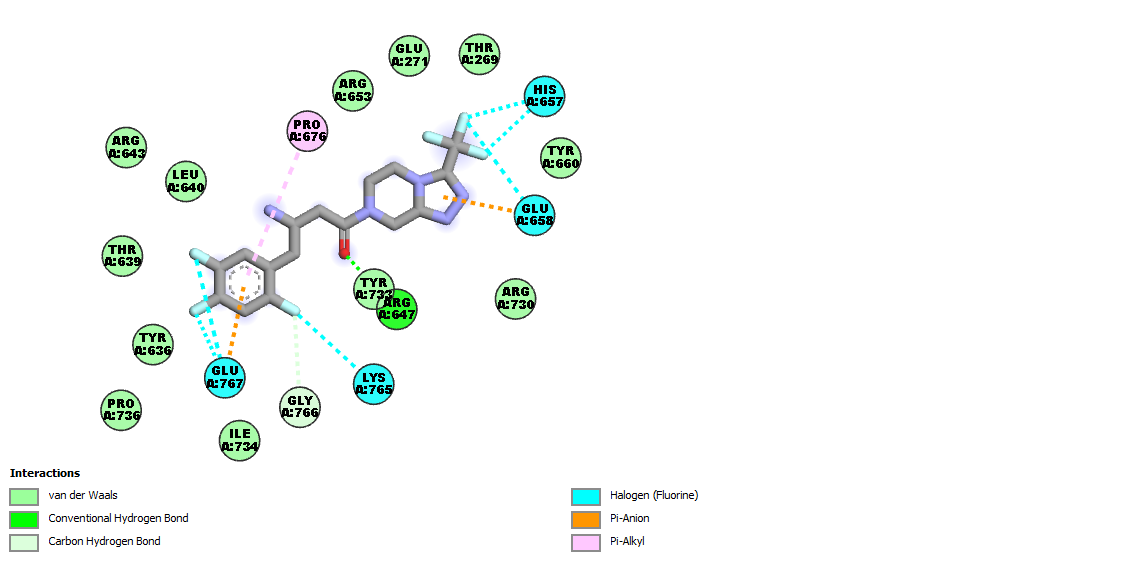

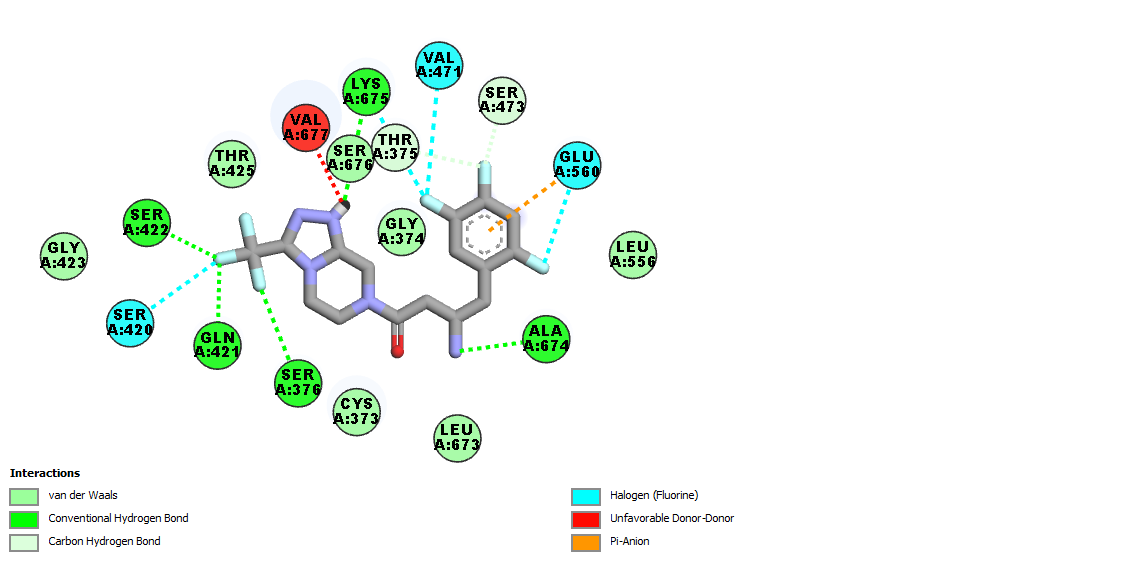

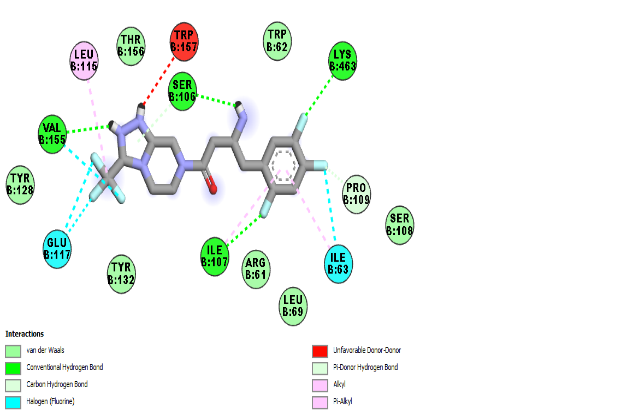

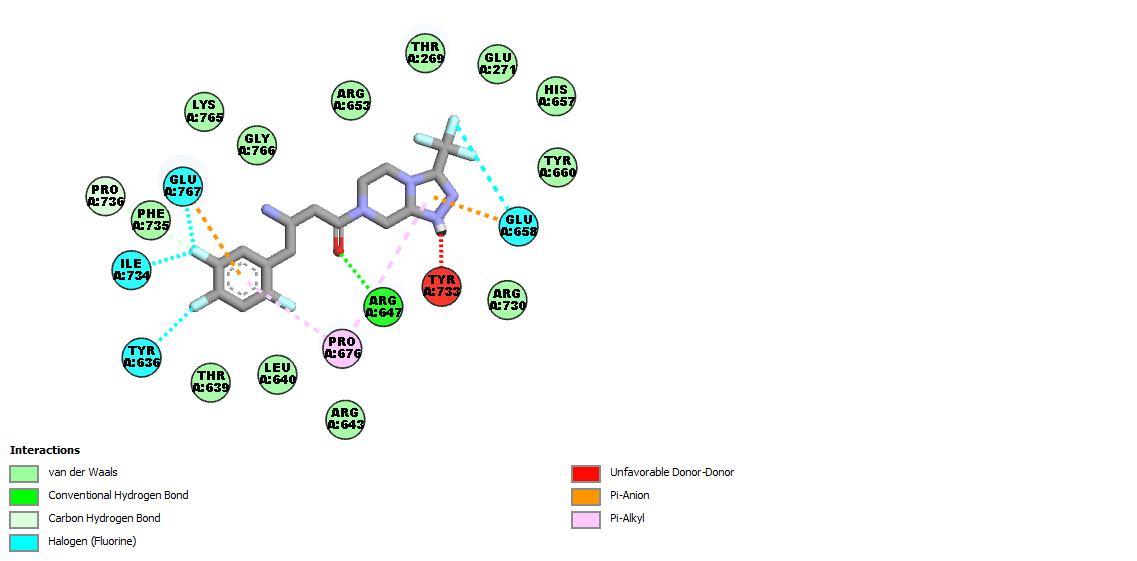

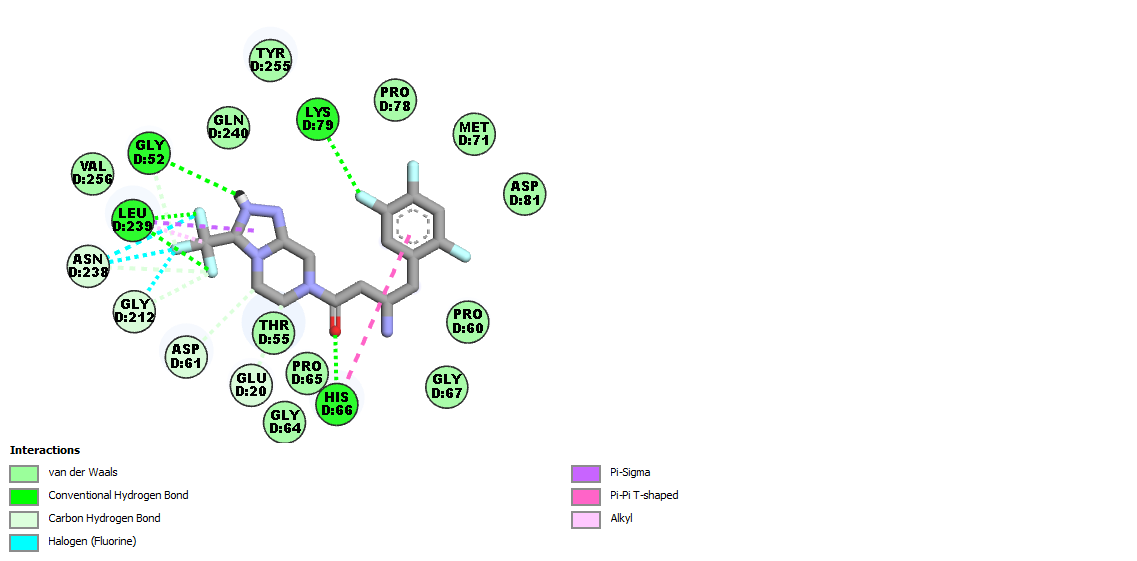

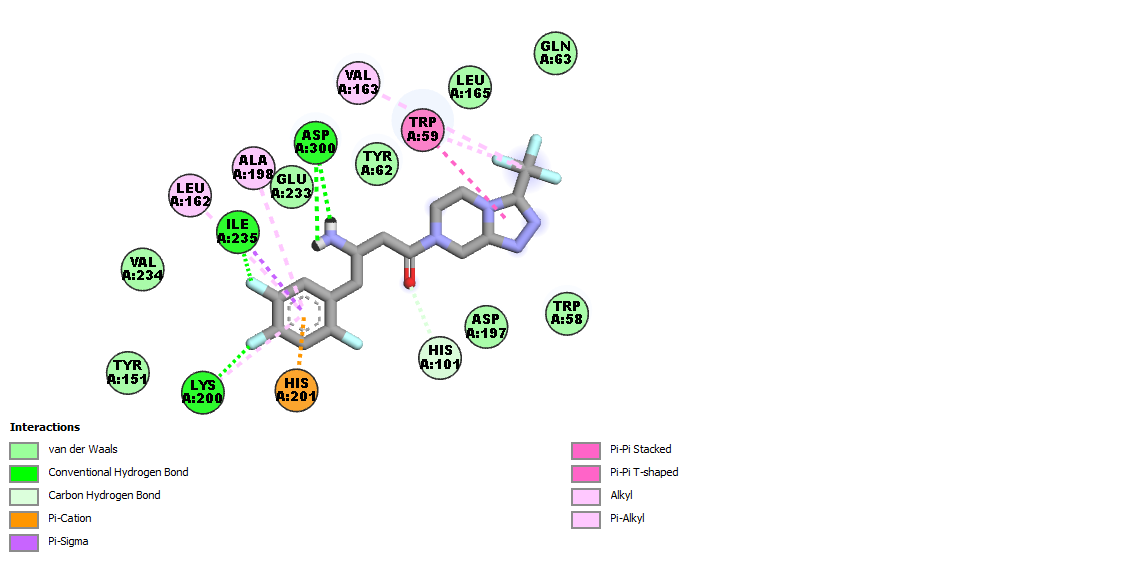

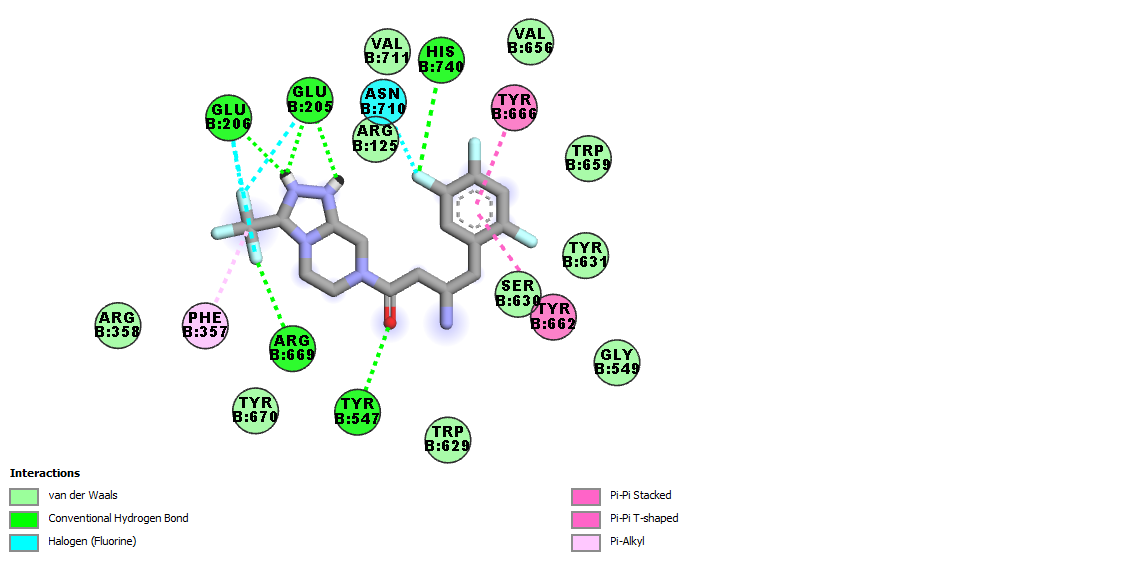

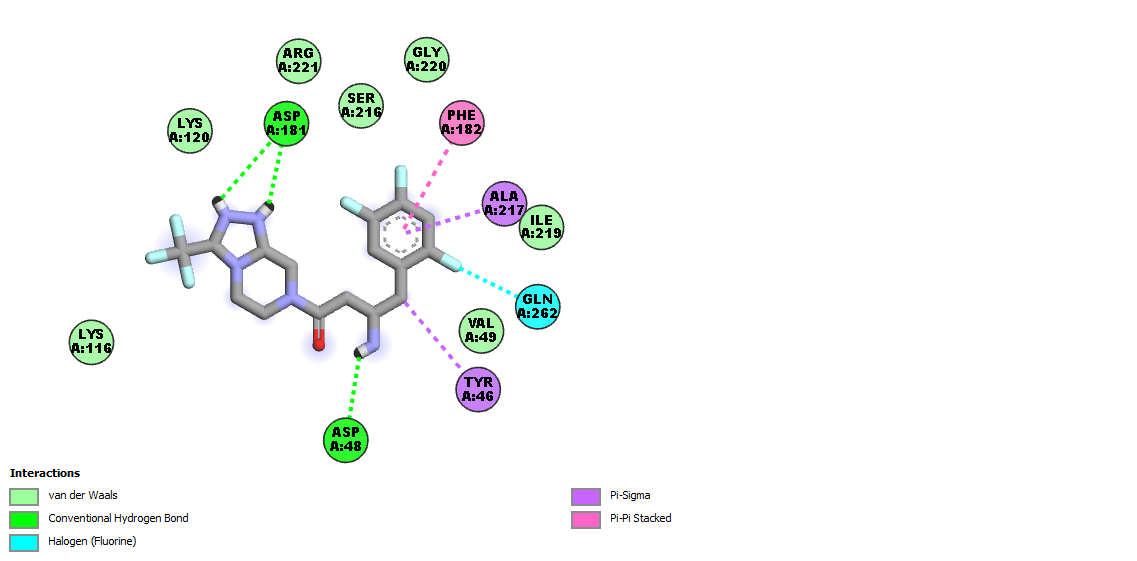


A

B

C

D

E

F

G

H

I

J

K

L

M

N

O

**
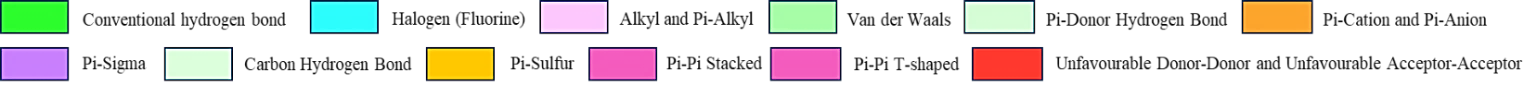
**

**Figure S2** Molecular docking interaction of Metformin with (**A**) 1FM9; (**B**) 1IR3; (**C**) 1V4S; (**D**) 1XU7; (**E**) 2HR7; (**F**) 2HWQ; (**G**) 2Q5S; (**H**) 2QMJ; (**I**) 2ZJ3; (**J**) 3C45; (**K**) 3CTT; (**L**) 3K35; (**M**) 3L2M; (**N**) 4A5S; (**O**) 4Y14.


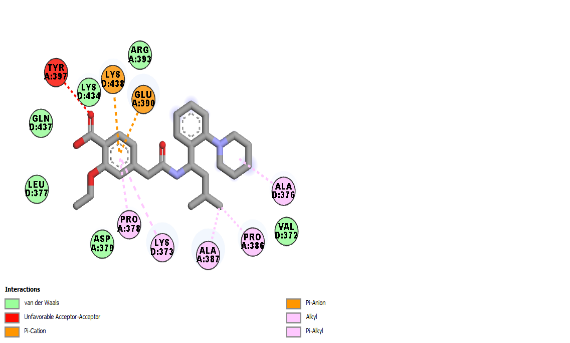

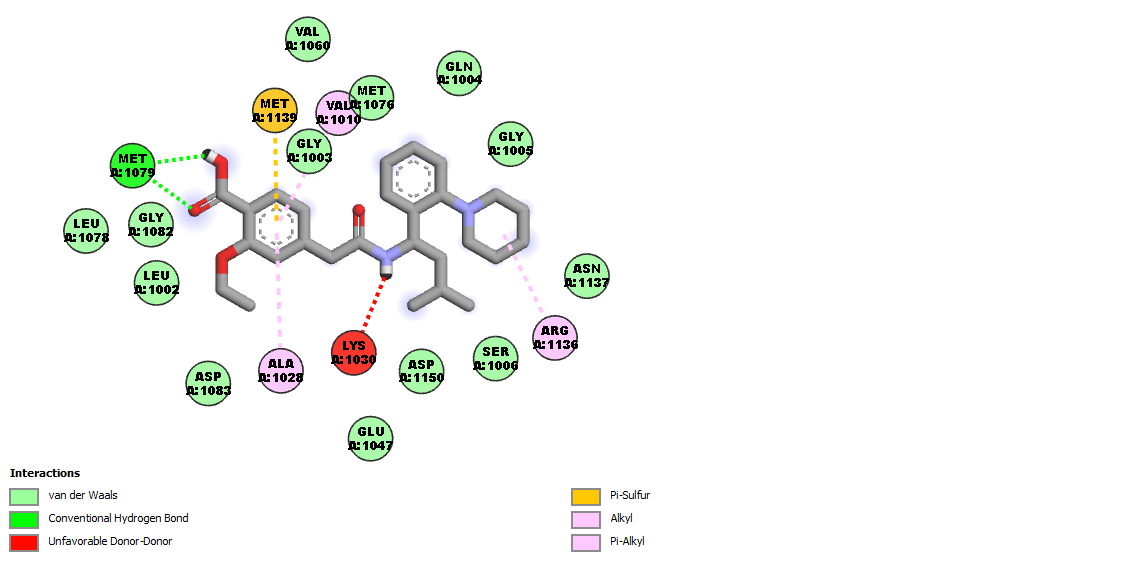

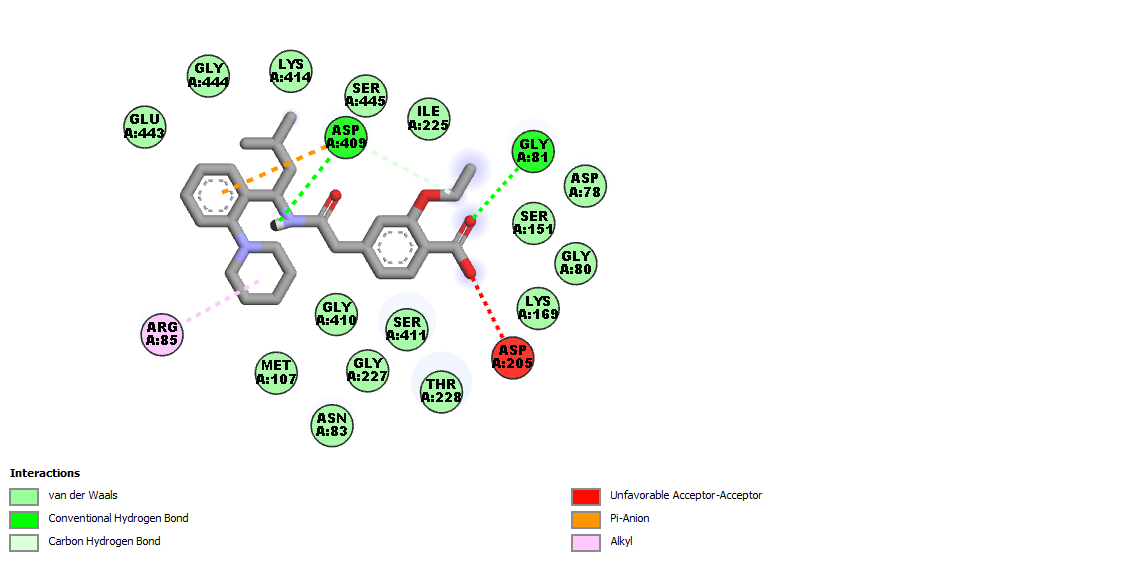

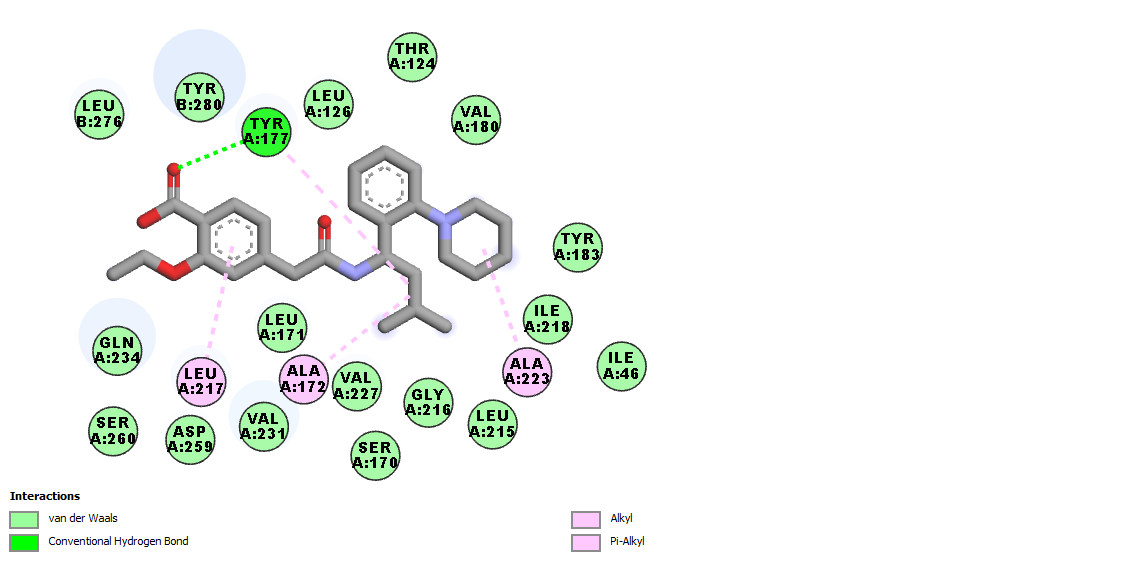

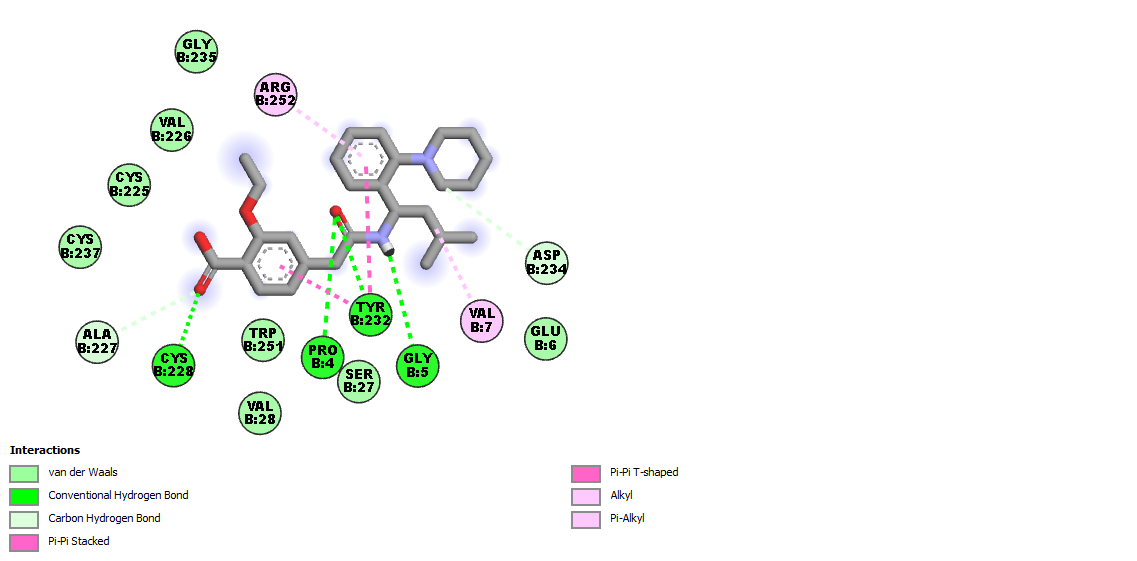

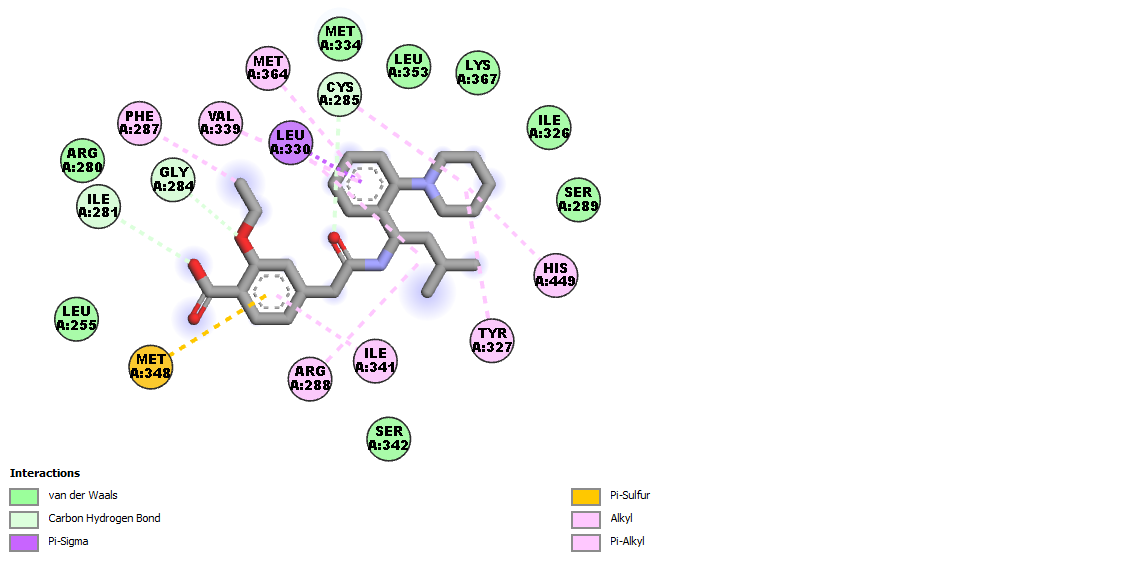

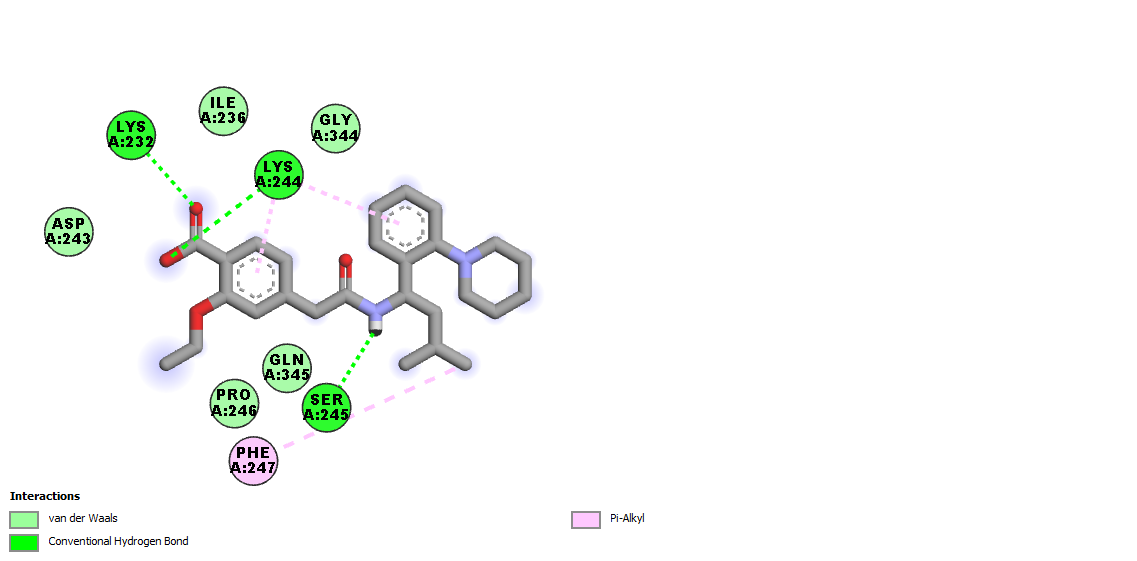

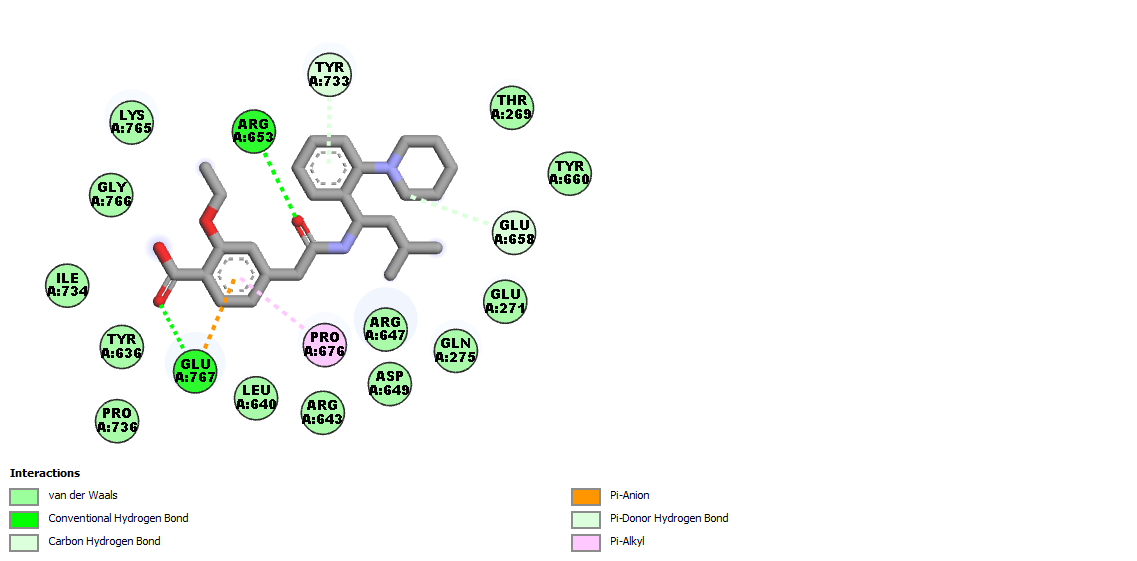

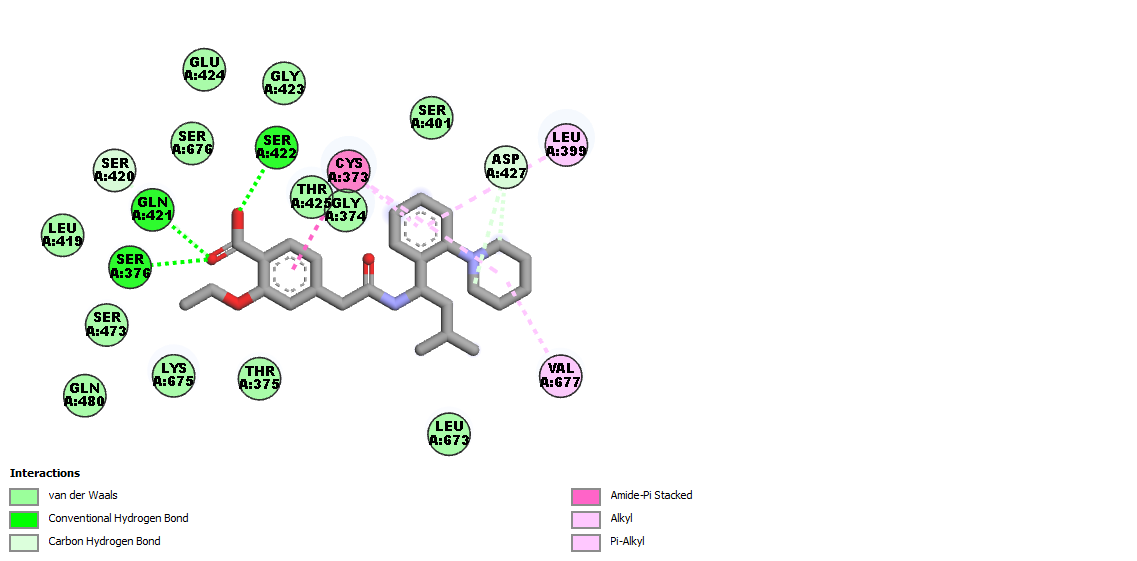

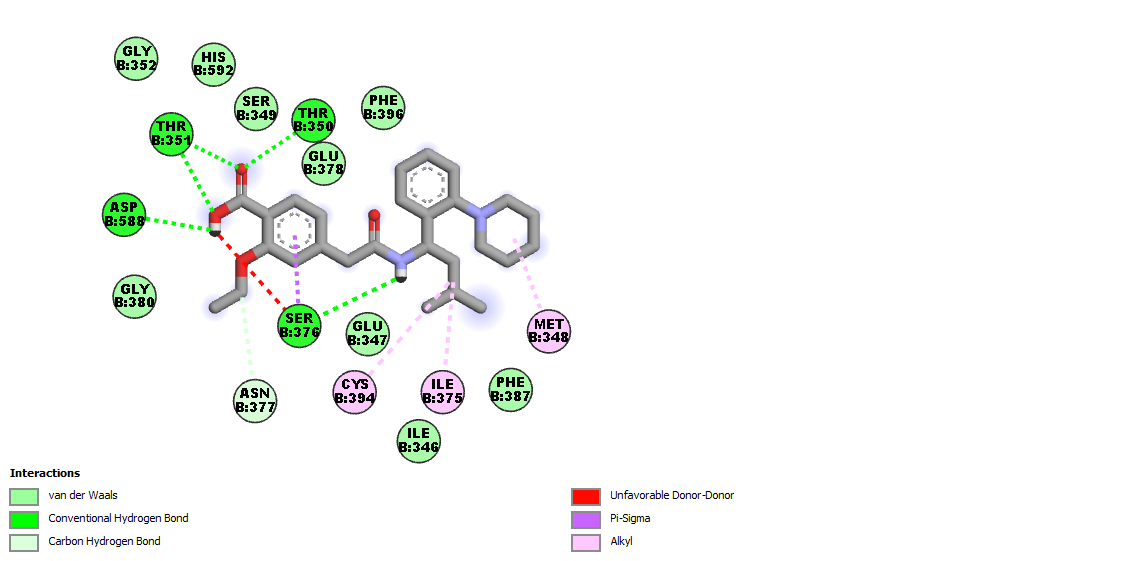

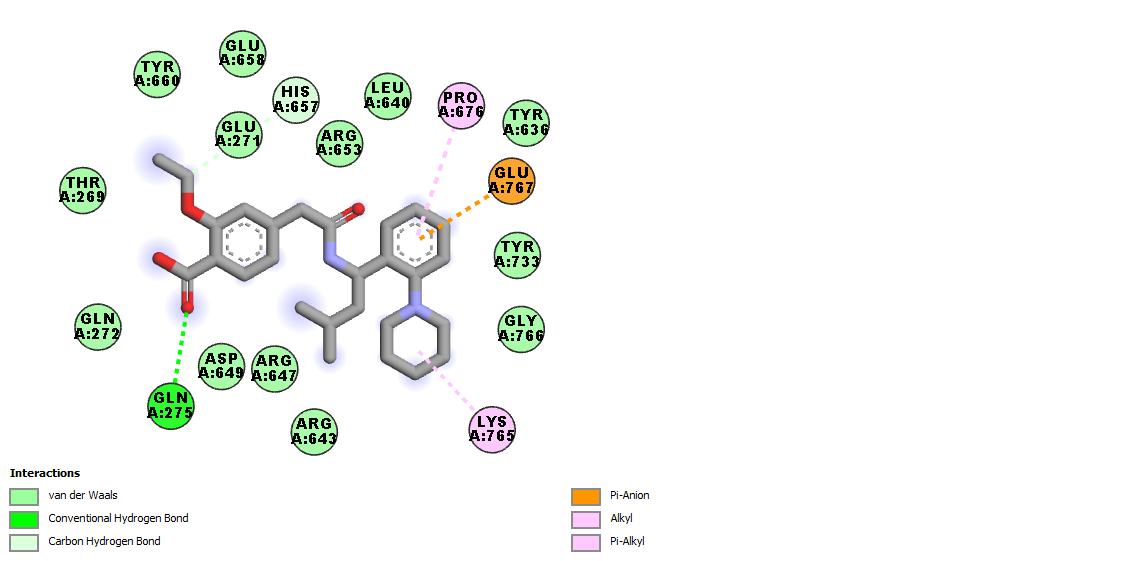

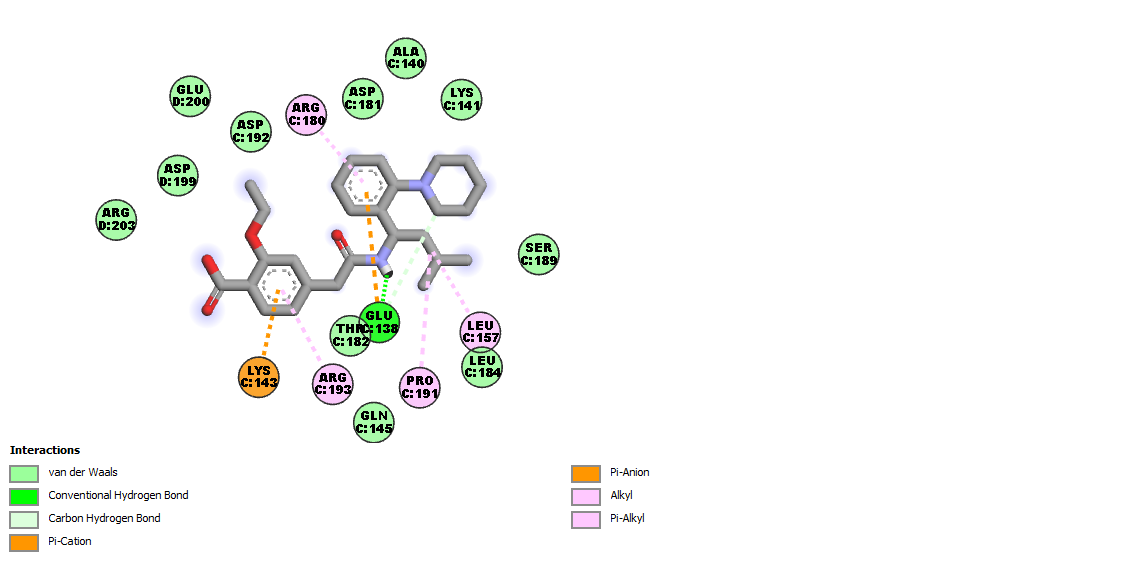

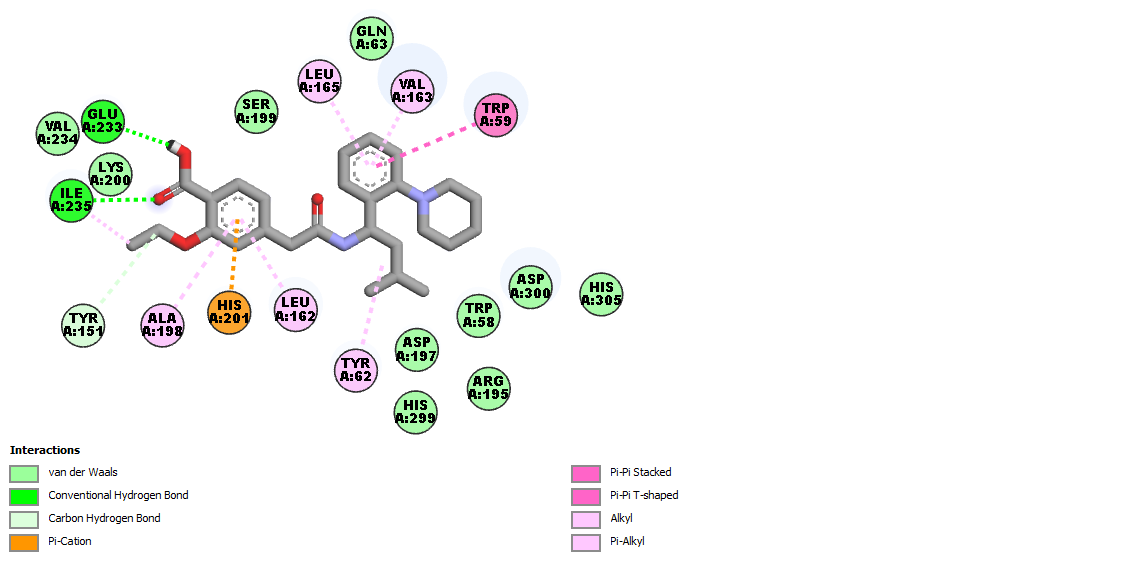

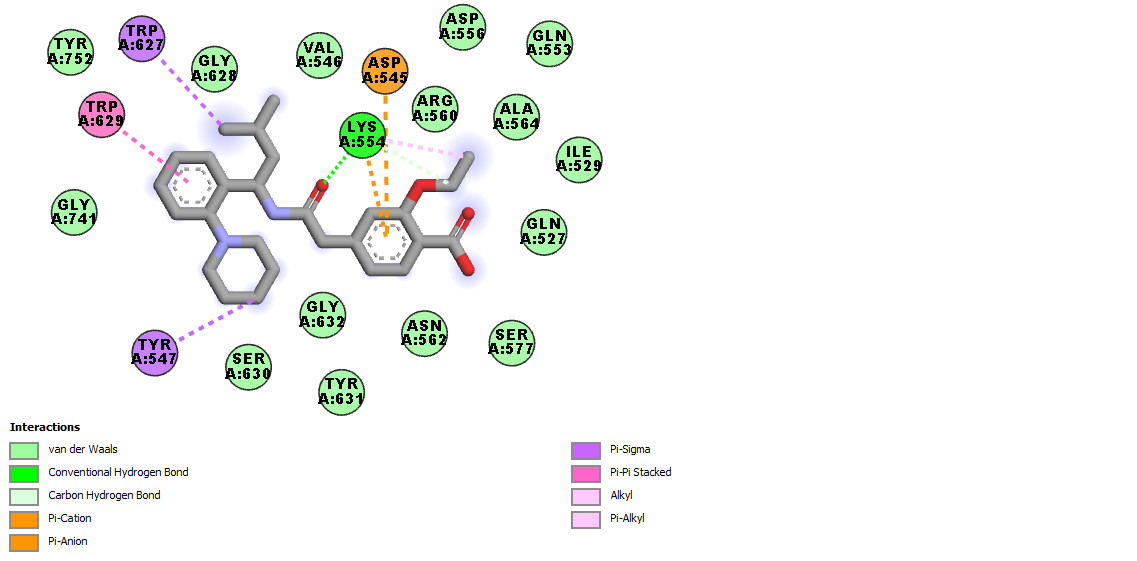

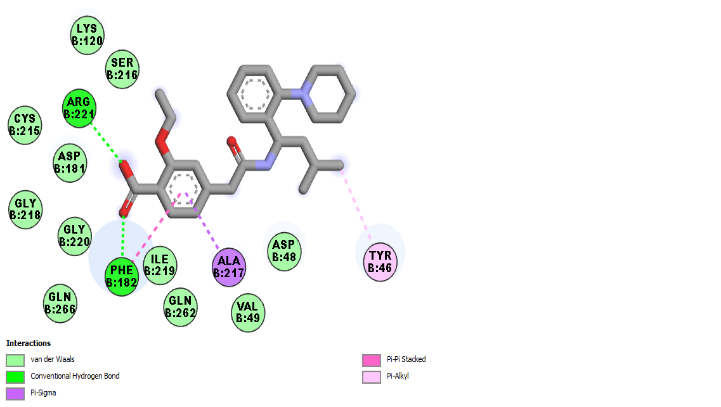


A

B

C

D

E

F

G

H

I

J

K

L

M

N

O

**
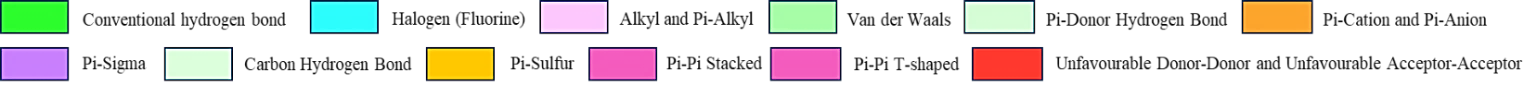
**

**Figure S3** Molecular docking interaction of Repaglinide with (**A**) 1FM9; (**B**) 1IR3; (**C**) 1V4S; (**D**) 1XU7; (**E**) 2HR7; (**F**) 2HWQ; (**G**) 2Q5S; (**H**) 2QMJ; (**I**) 2ZJ3; (**J**) 3C45; (**K**) 3CTT; (**L**) 3K35; (**M**) 3L2M; (**N**) 4A5S; (**O**) 4Y14.


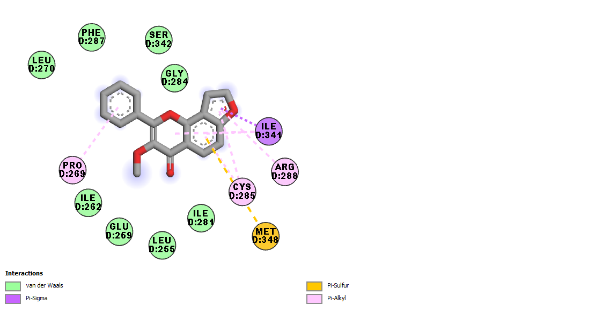

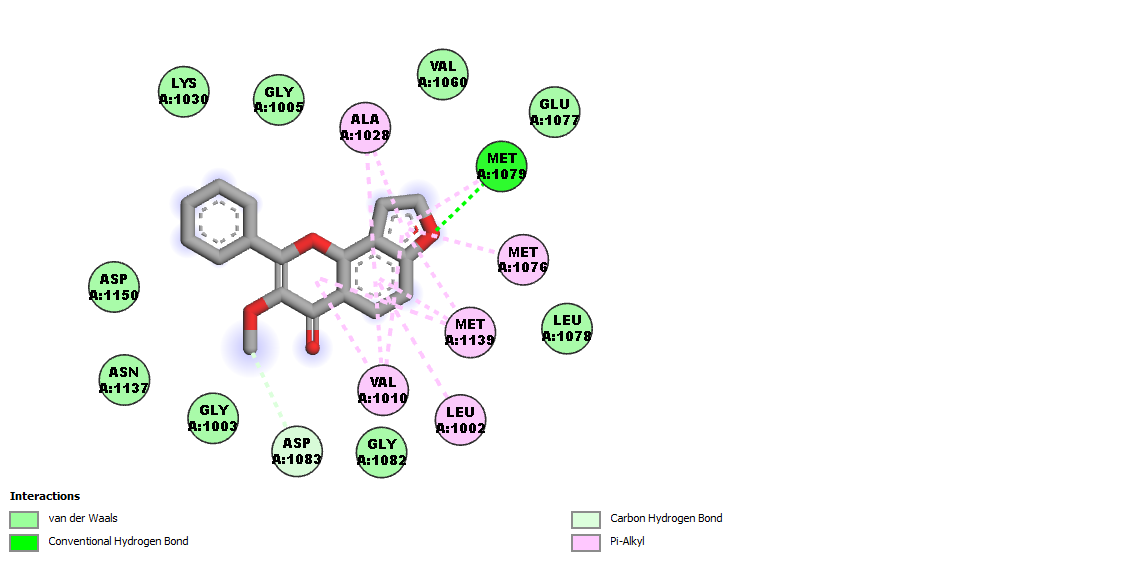

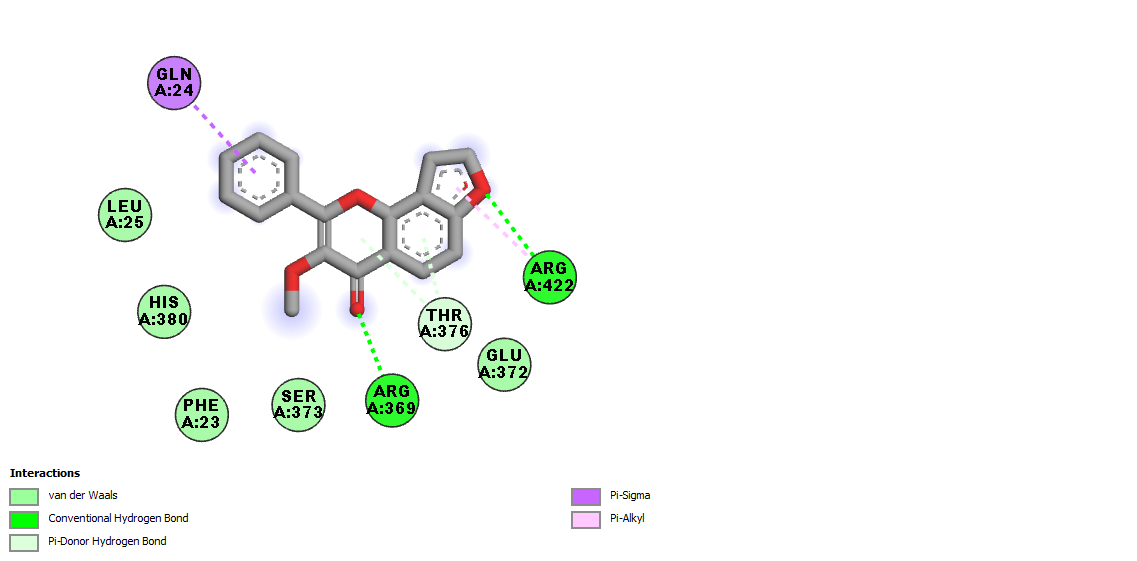

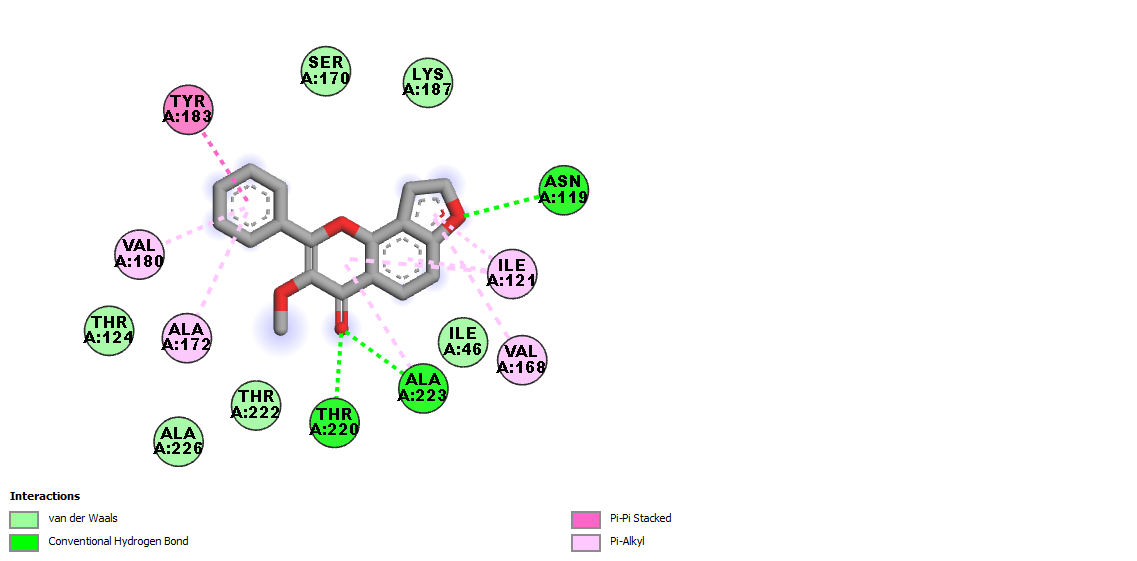

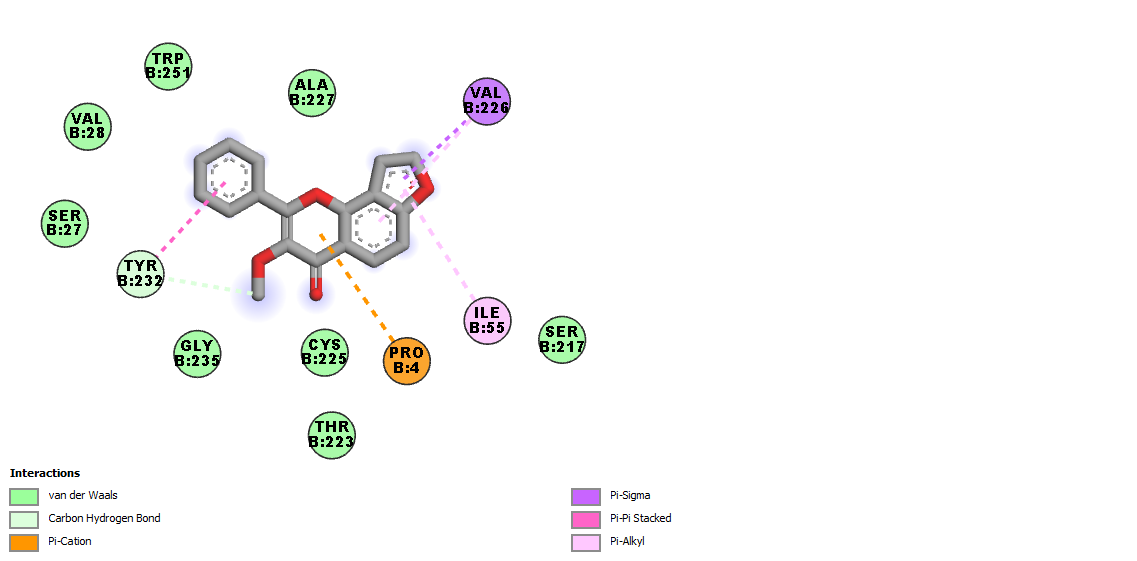

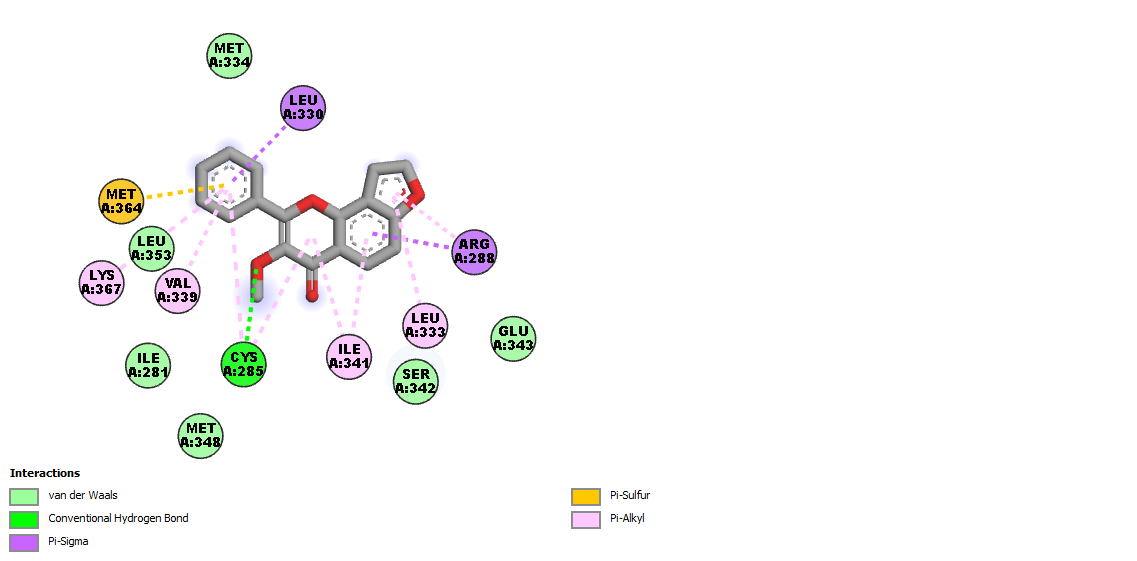

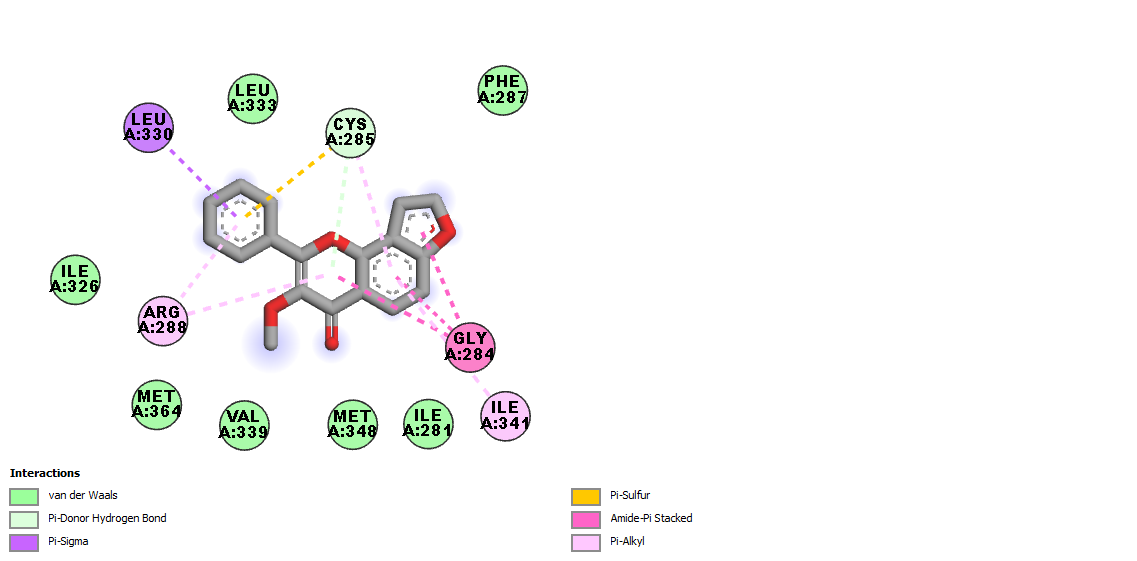

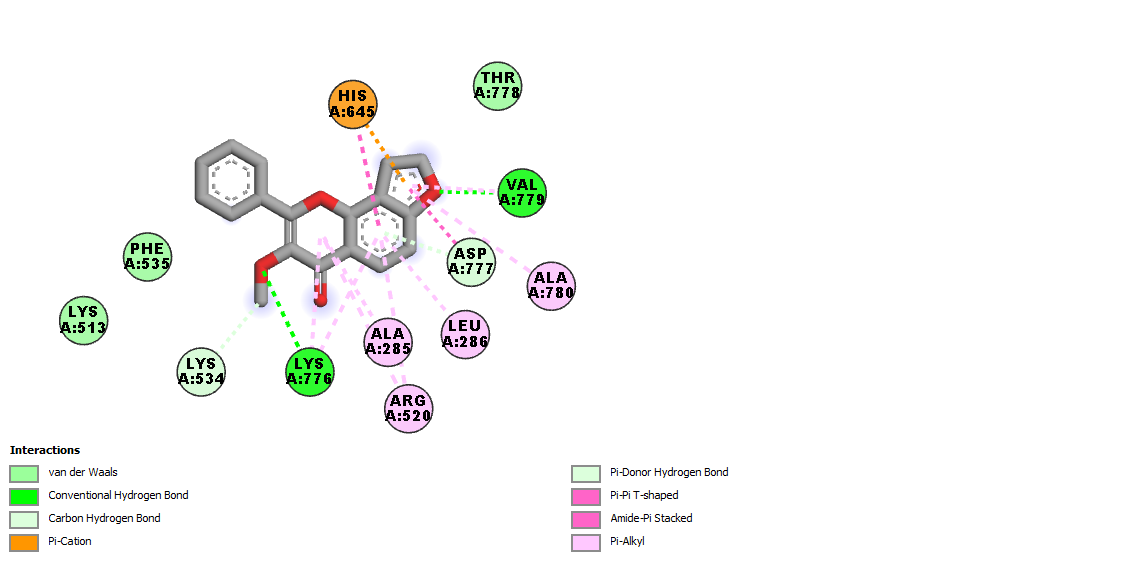

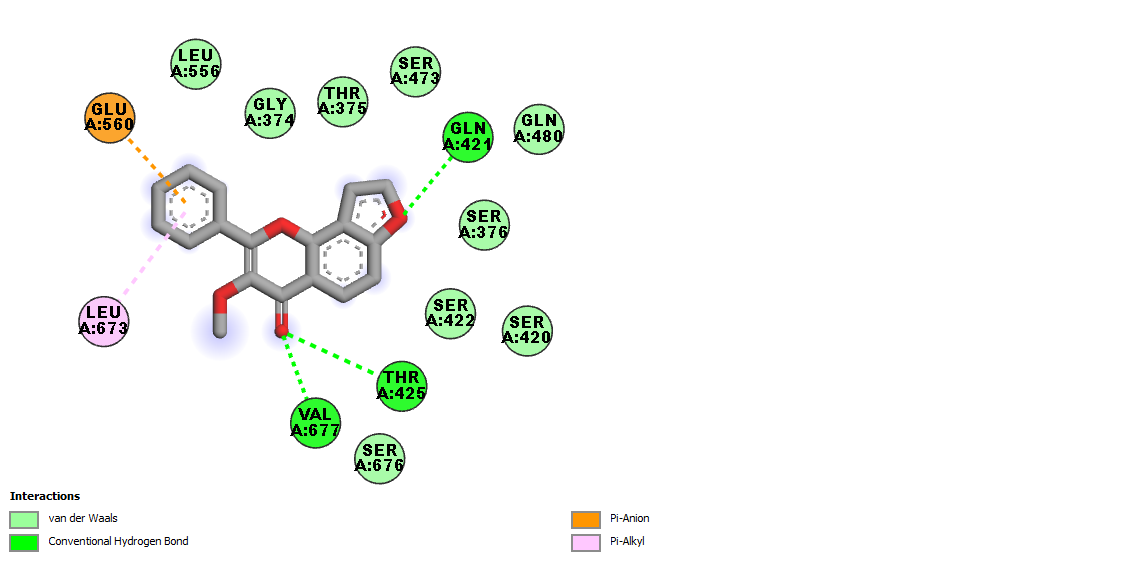

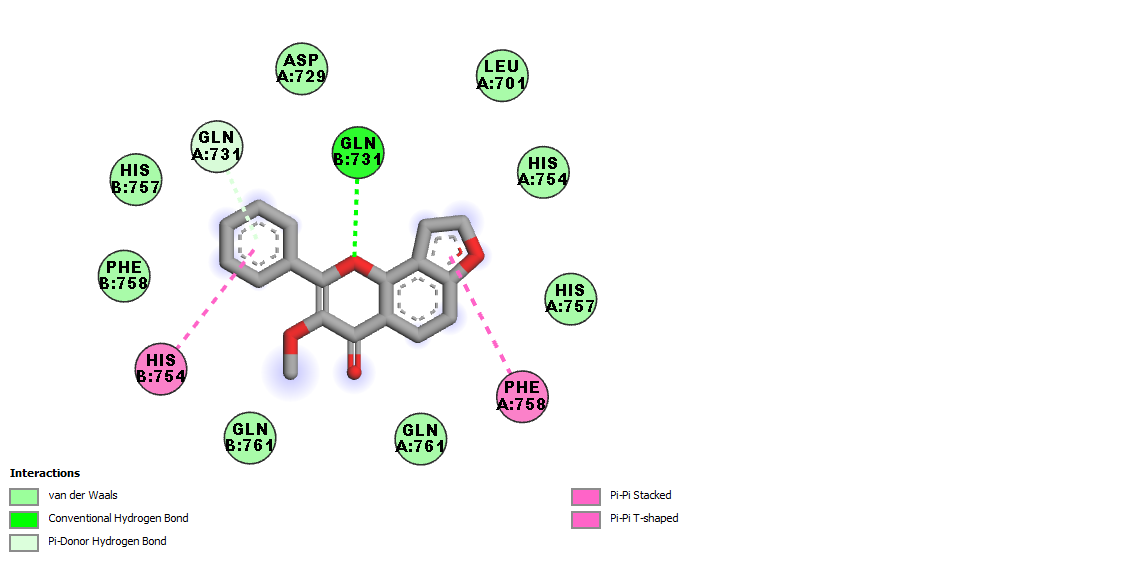

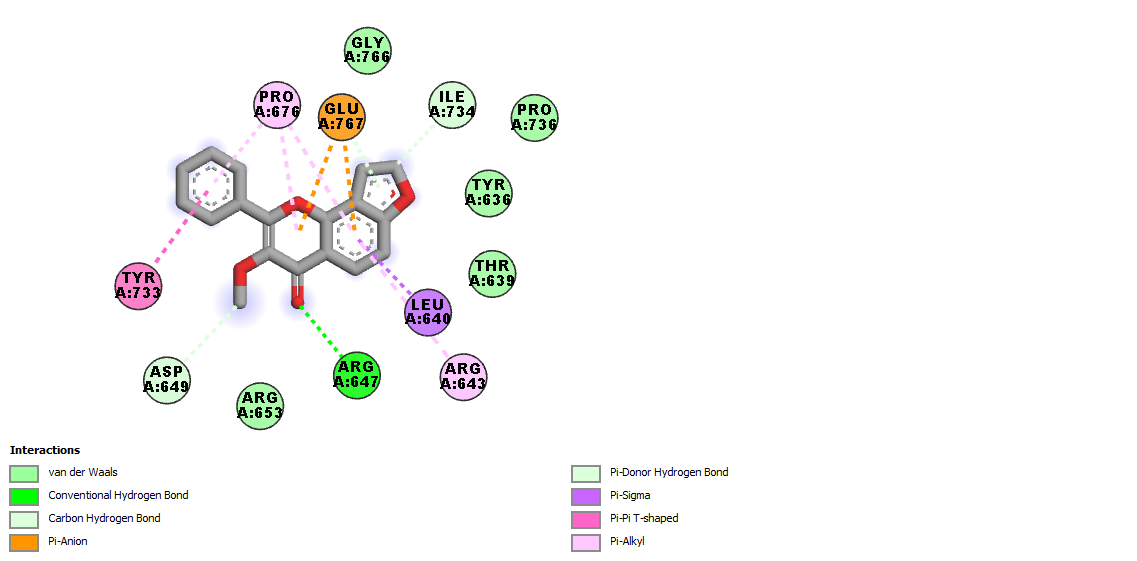

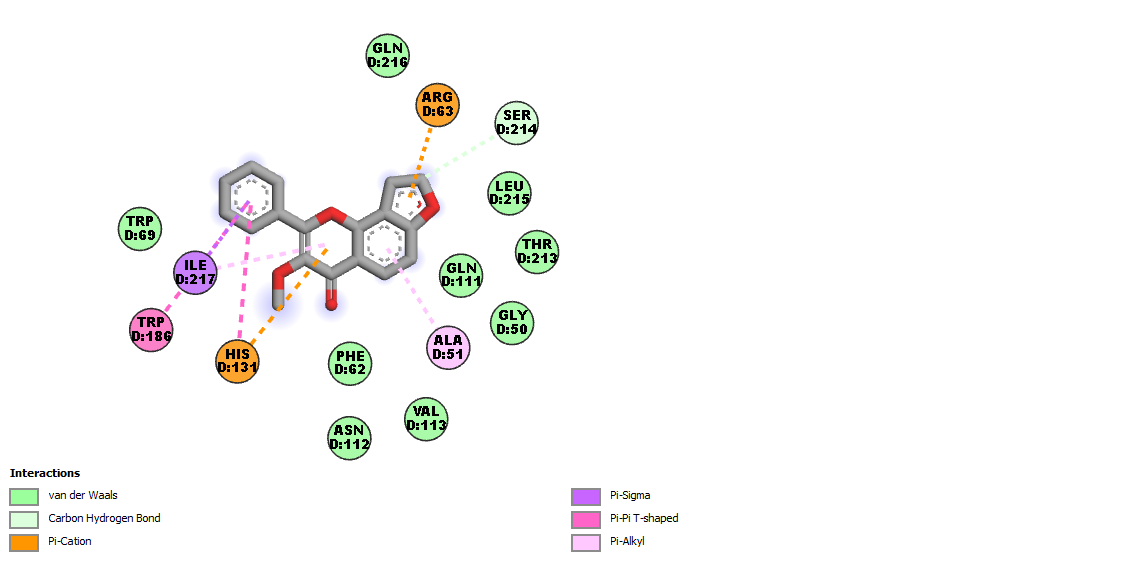

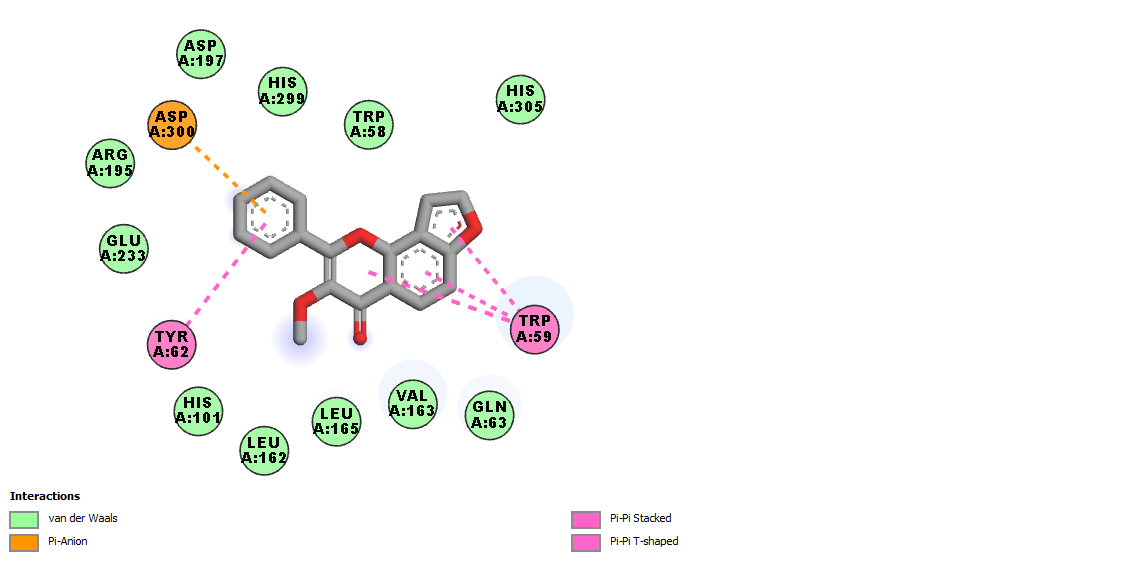

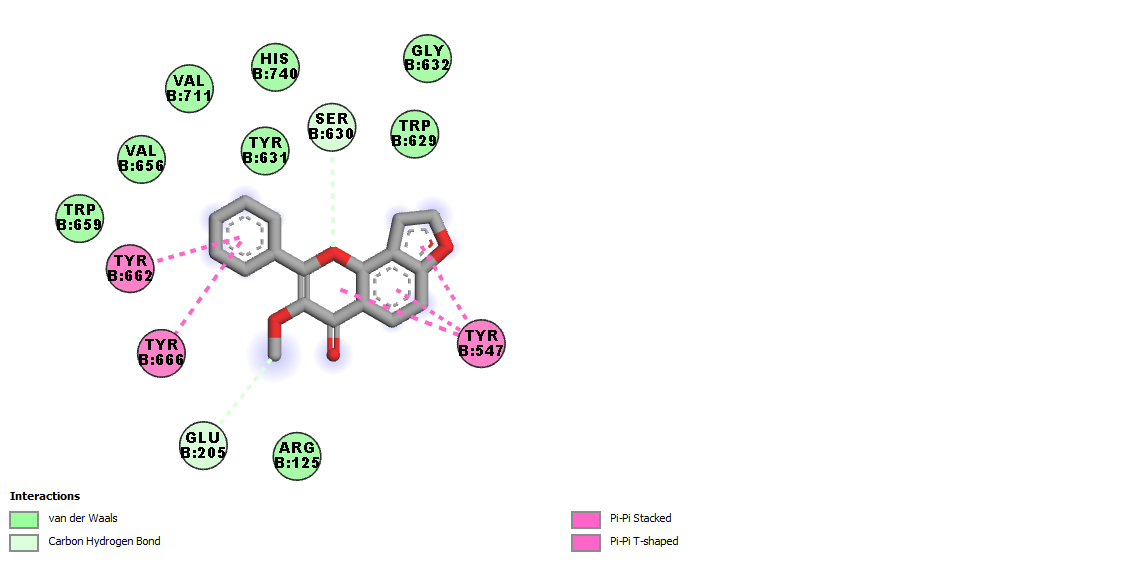

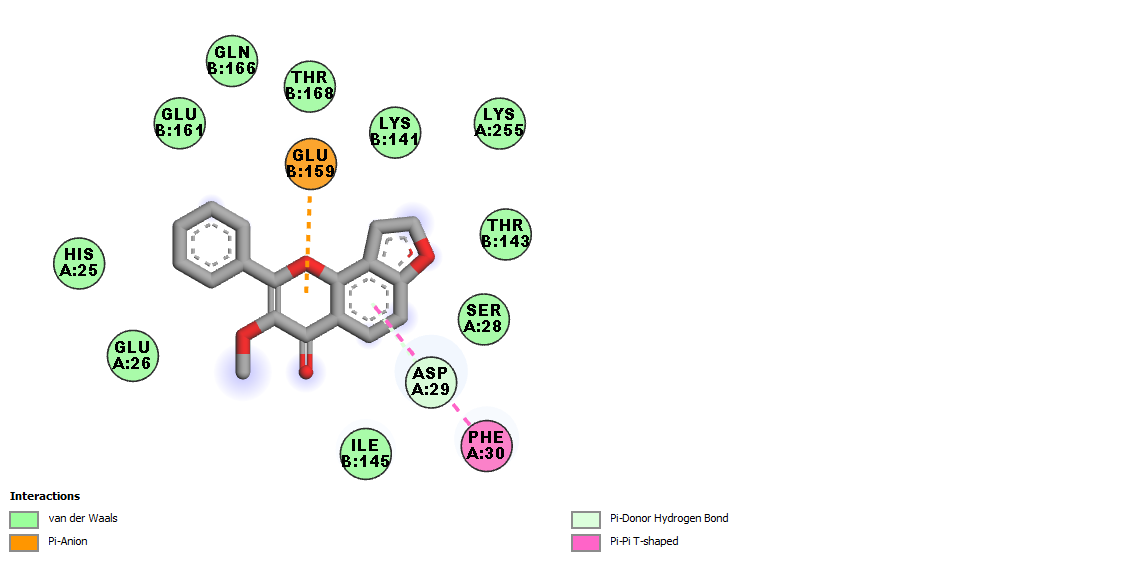


A

B

C

D

E

F

G

H

I

J

K

L

M

N

O

**
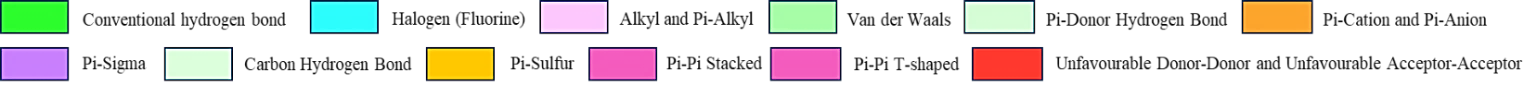
**

**Figure S4** Molecular docking interaction of Sitagliptin with (**A**) 1FM9; (**B**) 1IR3; (**C**) 1V4S; (**D**) 1XU7; (**E**) 2HR7; (**F**) 2HWQ; (**G**) 2Q5S; (**H**) 2QMJ; (**I**) 2ZJ3; (**J**) 3C45; (**K**) 3CTT; (**L**) 3K35; (**M**) 3L2M; (**N**) 4A5S; (**O**) 4Y14.
